# Supplementary material for: Do medical specialists accept claims-based Audit and Feedback for quality improvement? A focus group study
Source: BMJ Open. 2024 Apr 8;14(4):e081063. doi: 10.1136/bmjopen-2023-081063 (PMC11015254; doi:10.1136/bmjopen-2023-081063)
Supplement: Supplementary data [file bmjopen-2023-081063supp002.pdf]

# Supplementary file 2 – Description of Comparative Effectiveness Research studies

Comparative Effectiveness Research (CER) studies are conducted to compare the effectiveness or cost-effectiveness of two or more interventions (treatments or diagnostics) for a specific patient population, to identify which interventions work best for which patient. In a previous study (de Weerdt et al., 2023 currently under submission), we developed claims-based A&F for six CER studies conducted in the Netherlands. These CER studies are described below.

**Table. CER studies for which examples of claims-based A&F were developed**

| CER trial | Trial registration number* | Research question | Patient population | Intervention |
|---|---|---|---|---|
| 1) CAPP | NL9371 | What is the most (cost) effective treatment for children with complex appendicitis: operative or conservative treatment? | Children of 0-18yrs with complex appendicitis with/ without infiltrate/abscess | • Operative:<br>○ Laparoscopic<br>○ Open procedure<br>• Conservative |
| 2) Proclion | NA** | What is the most (cost) effective treatment for patients with critical ischemia of the legs: operative or conservative treatment? | Patients of ≥18yrs with critical ischemia of the legs | • Operative<br>○ Laparoscopic<br>○ Open procedure<br>• Conservative |
| 3) DART | NL6201 | What is the most (cost) effective treatment for elderly with an intra-articular distal radius fracture type C: operative or conservative treatment? | Patients of ≥65yrs with intra-articular distal radial fracture type C? | • Operative<br>• Conservative |
| 4) MIRA2 | NL7817 | What is the most effective treatment for woman with heavy menstrual bleeding: endometrial ablation or combined treatment of endometrial ablation with hormonal IUD? | Patients of 25-60yrs with heavy menstrual bleeding | • Endometrial ablation<br>• Endometrial ablation combined with hormonal IUD |
| 5) STONE | NL8128 | What is the most (cost) effective treatment for patients with obstructive kidney stones: Double J or Nephrostomy catheter? | Patients of ≥18yrs with obstructive kidney stones | • Double J catheter<br>• Nephrostomy catheter |
| 6) Growth hormone | NL6440 | What is the most (cost) effective treatment for children with idiopathic growth hormone deficiency that have normal growth hormone levels mid-puberty: continued growth hormone treatment or discontinued growth hormone treatment? | Patients of 10-18yrs with idiopathic growth hormone deficiency | • Continued growth hormone treatment<br>• Discontinued growth hormone treatment |

*from the Netherlands Trial Register
** Not registered due to observational study character
