# Supplementary material for: Do medical specialists accept claims-based Audit and Feedback for quality improvement? A focus group study
Source: BMJ Open. 2024 Apr 8;14(4):e081063. doi: 10.1136/bmjopen-2023-081063 (PMC11015254; doi:10.1136/bmjopen-2023-081063)
Supplement: Supplementary data [file bmjopen-2023-081063supp003.pdf]

Supplementary File 3 - Focus group protocol

At the beginning of each focus group rules of conduct were set, the aim of the focus group was explained, and informed consent was repeated. Prior to the start of the actual content of the focus group all researchers and participants were asked to introduce themselves and to describe their current employment.

The main research question was:

What are the perceptions of medical specialists on claims-based Audit & Feedback for QI?

The following sub-questions were discussed during the focus groups:

(1) Are you familiar with A&F and/or with claims-based A&F? What are your perceptions on A&F and/or claims-based A&F?

Participants were presented with visual examples of claims-based A&F for Comparative Effectiveness Research (CER) study.

(2) What are your perceptions on this example of claims-based A&F?

(3) Would you accept this claims-based A&F on CER implementation status?
