# Supplementary material for: Do medical specialists accept claims-based Audit and Feedback for quality improvement? A focus group study
Source: BMJ Open. 2024 Apr 8;14(4):e081063. doi: 10.1136/bmjopen-2023-081063 (PMC11015254; doi:10.1136/bmjopen-2023-081063)
Supplement: Supplementary data [file bmjopen-2023-081063supp004.pdf]

Supplementary Files

Claims-based A&F on CER implementation, as shown during focus groups

CER study: DART

CER research question: What is the most (cost)effective treatment for patients ≥65 years old with an intra-articular radial fracture type C: conservative or operative treatment?

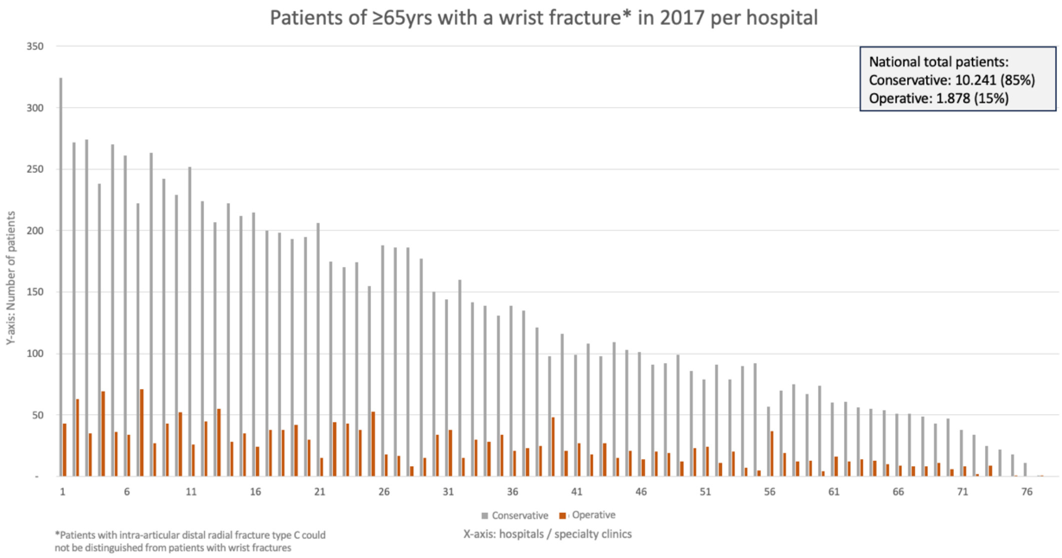

de Weerdt V, *et al. BMJ Open* 2024; 14:e081063. doi: 10.1136/bmjopen-2023-081063

CER study: STONE

CER research question: What is the most (cost) effective treatment for patients with obstructive kidney stones: Double J or Nephrostomy catheter?

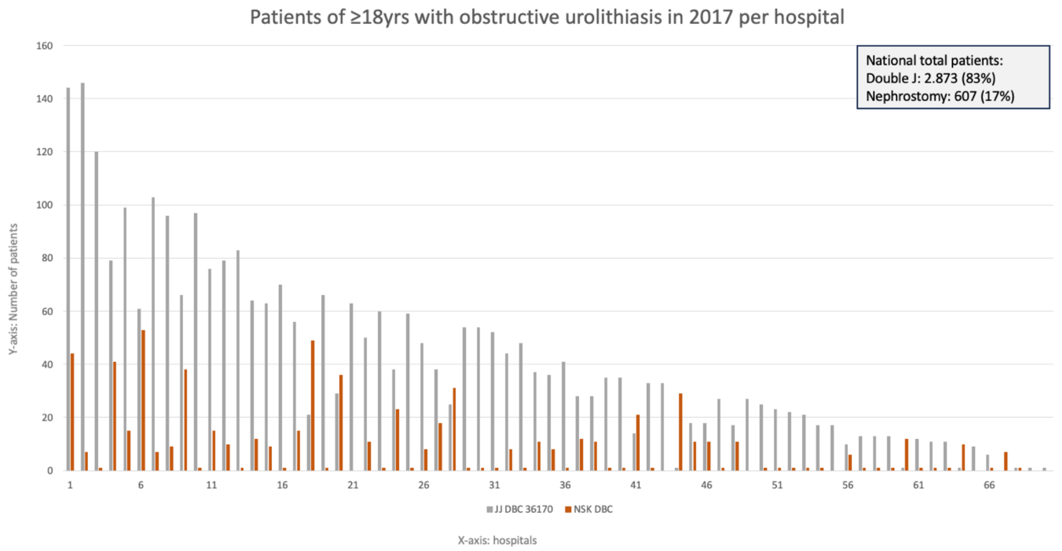

de Weerdt V, *et al. BMJ Open* 2024; 14:e081063. doi: 10.1136/bmjopen-2023-081063

CER study: Proclion

CER research question: What is the most (cost) effective treatment for patients with critical ischemia of the legs: operative or conservative treatment?

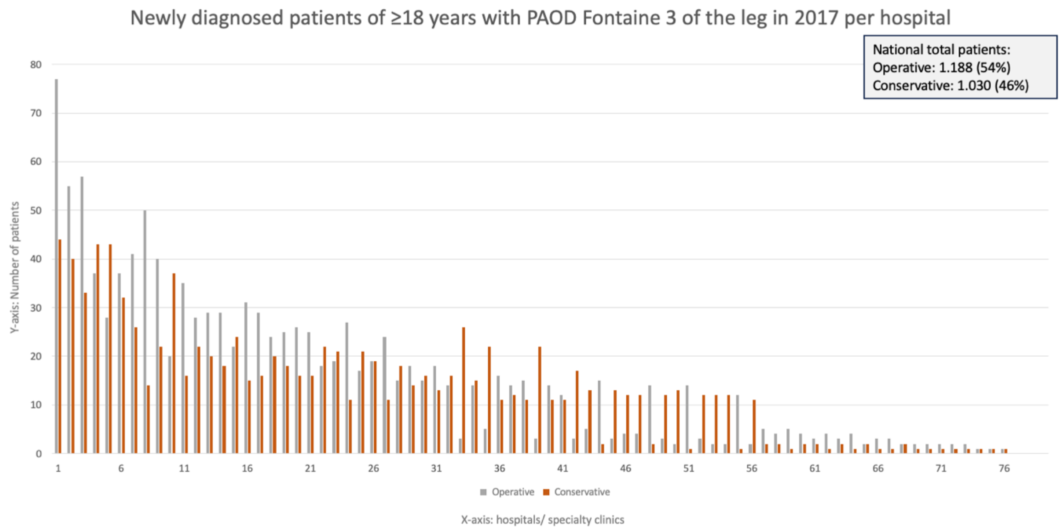

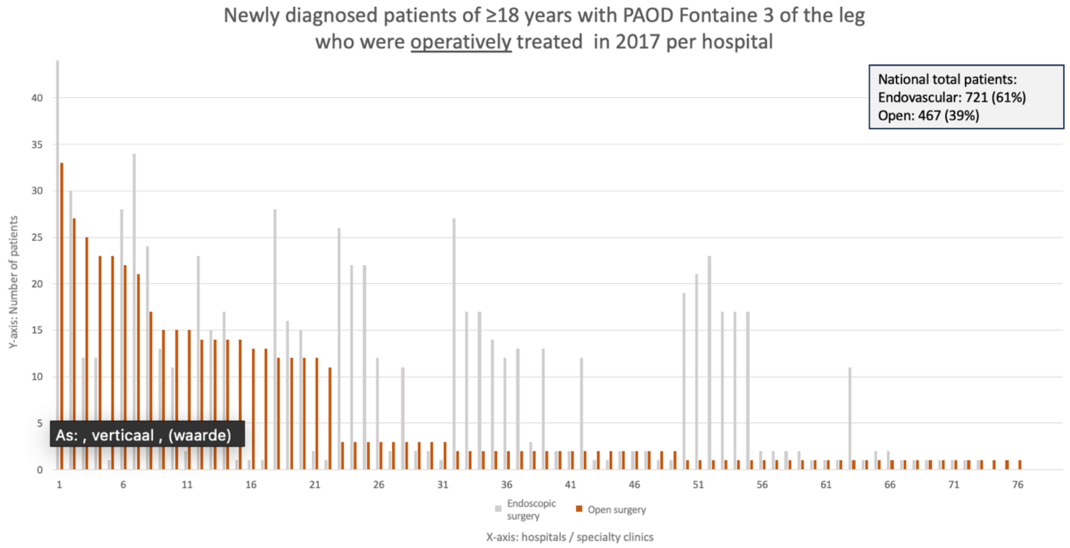

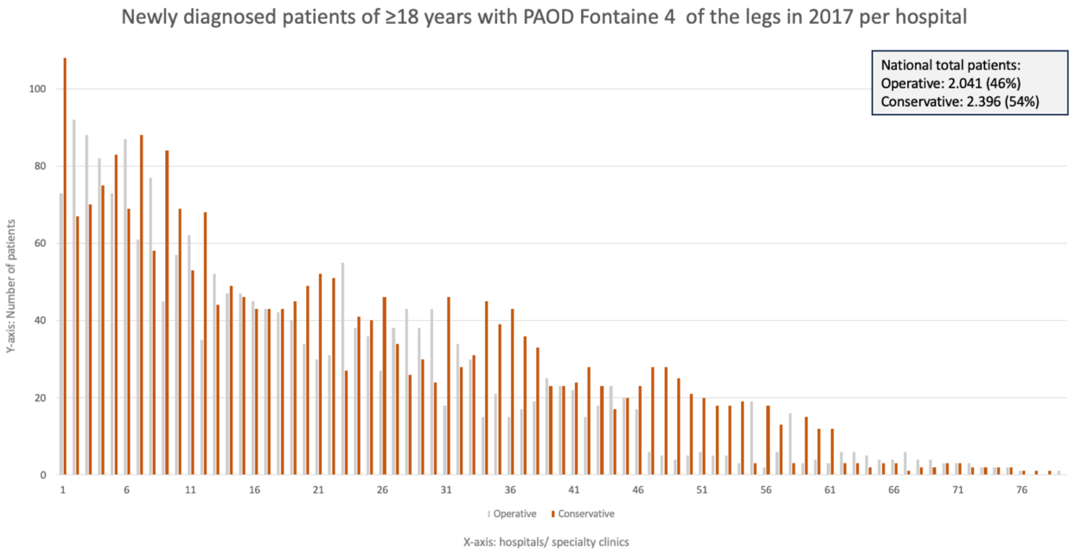
Newly diagnosed patients of ≥18 years with PAOD Fontaine 4 of the legs in 2017 per hospital
National total patients:
Operative: 2.041 (46%)
Conservative: 2.396 (54%)
Y-axis: Number of patients
Operative
Conservative
X-axis: hospitals/ specialty clinics

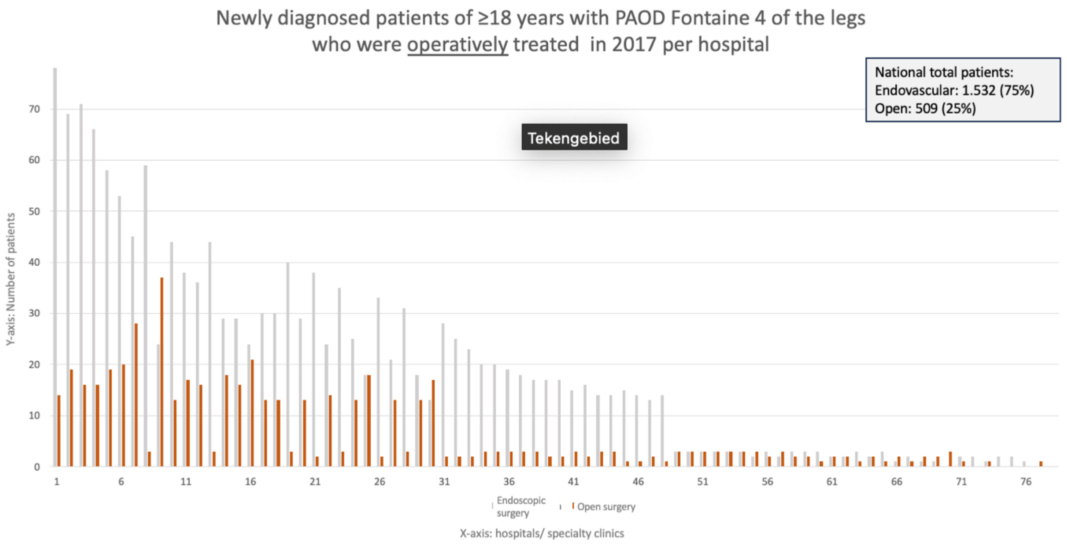
Newly diagnosed patients of ≥18 years with PAOD Fontaine 4 of the legs
who were operatively treated in 2017 per hospital
National total patients:
Endovascular: 1.532 (75%)
Open: 509 (25%)
Tekengebied
Y-axis: Number of patients
Endoscopic surgery
Open surgery
X-axis: hospitals/ specialty clinics

CER study: MIRA2

CER research question: What is the most (cost) effective treatment for woman with heavy menstrual bleeding: endometrial ablation or combined treatment of endometrial ablation with hormonal IUD?

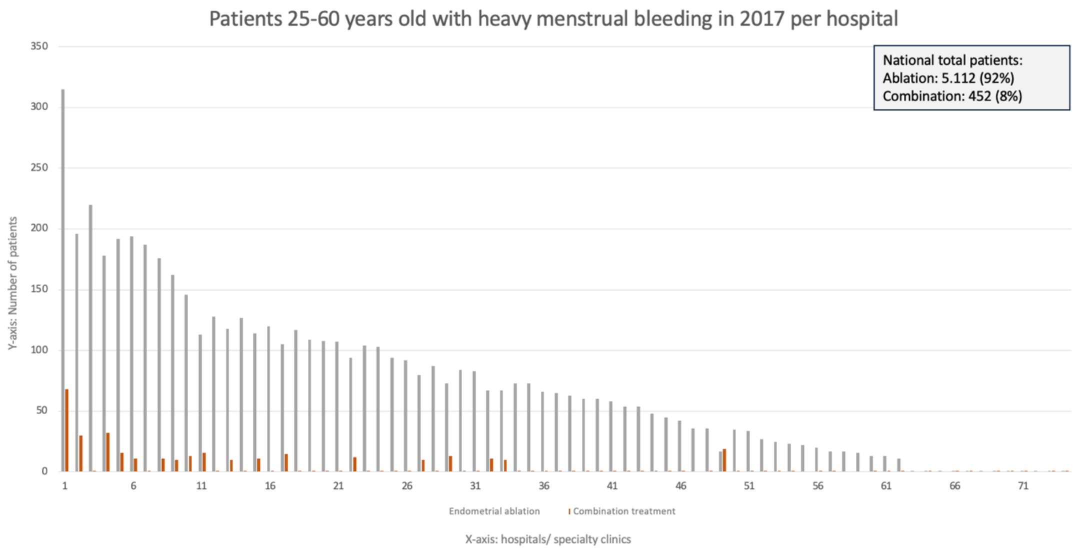

de Weerdt V, *et al. BMJ Open* 2024; 14:e081063. doi: 10.1136/bmjopen-2023-081063

CER study: CAPP

CER Research Question: What is the most (cost) effective treatment for children with complex appendicitis?

- Is laparoscopic or open appendectomy most (cost) effective for children <18 years old with complex appendicitis without abscess/mass formation?
- Is conservative or operative treatment most (cost) effective for children <18 years old with complex appendicitis with abscess/mass formation?

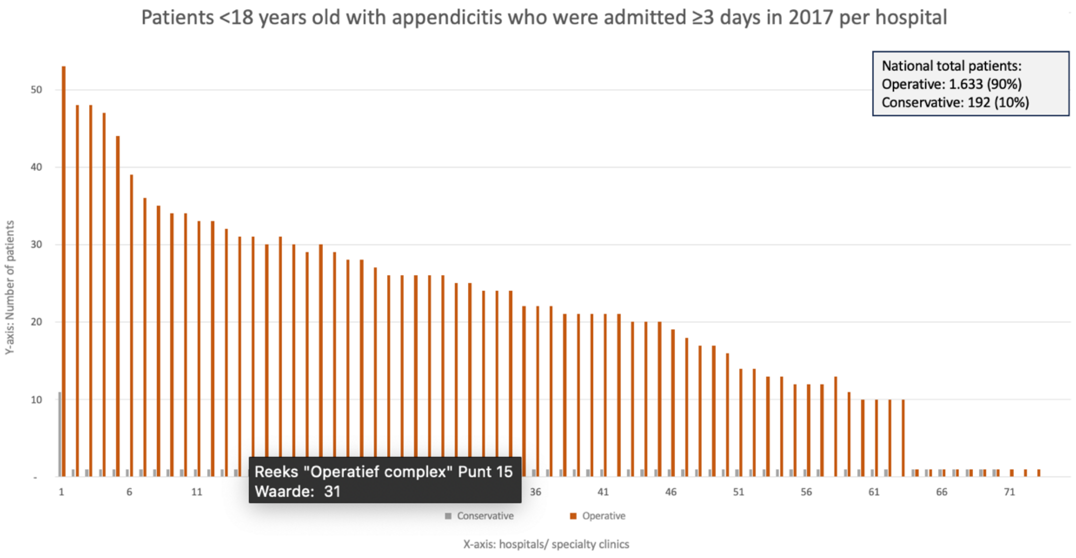

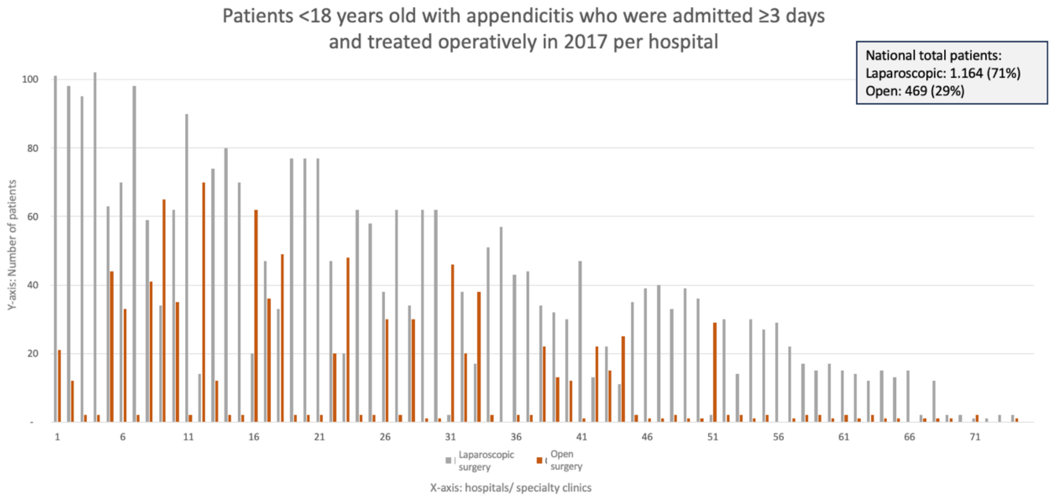

de Weerdt V, *et al. BMJ Open* 2024; 14:e081063. doi: 10.1136/bmjopen-2023-081063
