# Supplementary material for: Do medical specialists accept claims-based Audit and Feedback for quality improvement? A focus group study
Source: BMJ Open. 2024 Apr 8;14(4):e081063. doi: 10.1136/bmjopen-2023-081063 (PMC11015254; doi:10.1136/bmjopen-2023-081063)
Supplement: Supplementary data [file bmjopen-2023-081063supp005.pdf]

Table 2: Participants attitudes towards A&F

Focus group	Participant	Attitude towards A&F*	Attitude towards claims-based A&F	Attitude towards A&F developed for this CER study
**DART**	D1	+/-	+/-	-
	D2	+/-	+/-	NS
	D3	+	-	-
	D4	+	+/-	NS
	D5	+	+	NS
	D6	+/-	+/-	+/-
	D7	+/-	+/-	-
**STONE**	S1	+	+	+
	S2	-	-	+/-
	S3	+	+	+
	S4	NS	NS	-
**Proclion**	P1	+/-	-	-
	P2	+/-	-	-
**MIRA2**	M1	+	+/-	-
	M2	+	NS	-
	M3	NS	-	-
	M4	+	NS	-
	M5	+	-	-
**CAPP**	C1	+	+/-	+/-
	C2	+	+/-	+
	C3	+	+	+

* (+)=positive attitude, (+/-)=mixed attitude, (-)=negative attitude, NS=not specified
