# Supplementary material for: Do medical specialists accept claims-based Audit and Feedback for quality improvement? A focus group study
Source: BMJ Open. 2024 Apr 8;14(4):e081063. doi: 10.1136/bmjopen-2023-081063 (PMC11015254; doi:10.1136/bmjopen-2023-081063)
Supplement: Supplementary data [file bmjopen-2023-081063supp006.pdf]

Table 3. Results discourse analysis

		Quotes	Keywords or Figures of speech	Discourse	Argument
In favour of claims-based A&F	1.1	*"I think this (claims-based A&F) is a visual attractive tool to set everything in motion (…) and to stimulate implementation of result results, I do think it helps"*	Sets in motion	A&F gives insight in own performance and thereby creates action	1.1 A&F stimulates reflective learning and improvement
		"A&F is useful to start a conversation for change, A&F is the first step to initiate change"	Start<br>First step		
		"Showing people a mirror is why it works"	Comparison of A&F to a mirror		
	1.2	*"If you can get it out of claims data, then you have objective data on your own process, because otherwise it is dependent on what the person registers and that can be less reliable"*	Objective data	Claims-data is more reliable than self-reported data, thus claims-based A&F is more reliable than other types of A&F	1.2 Claims-based A&F is more reliable than other types of A&F
		"Claims data can give a more reliable portrayal of clinical practice, thus then you get reliable A&F: now it is dependent on the administrator whether A&F is accurate, if you can enhance that through A&F based on data of other sources, then you have a better and more accurate registration which gives better A&F"	Reliable<br>Accurate		
Opposing claims-based A&F	2.1	*"I wonder then if you would have the same data and would show it to hospitals if that would really lead to change of practice. I don't know yet. Maybe they will still think of reasons to deviate from the norm"*	Wonder if that would lead to change	Questioning the effect of claims-based A&F	2.1 A&F is insufficient to create change of clinical behaviour
		"If you get the A&F, you need to act upon it, but you can't reinforce that, so… there is the dilemma: you can show it, but subsequently you want change, but that change depends on individual hospitals, so…".	Can't reinforce (…) action<br>Dilemma	Questioning the effect of claims-based A&F	
		"If you have the feeling you are doing evidence-based treatments and that all it takes is to register claim codes differently, then you will change claims codes, we are not more catholic than the pope"	Change claims codes<br>Metaphor: not more catholic than the pope	Posing claims-based A&F leads to different registration of claims-codes, not change of clinical behaviour	

2.2	[when shown example of claims-based A&F] *"Yes, it is a nice overview. Only, ehm yes, what are the consequences of this figures? I can't .. ehm.. I can't see how to translate this to my practice? [...] How do we or the patient benefit from this exactly?".*	How do we or the patient benefit?	Questioning the relevance of the A&F	2.2 Claims-based A&F lacks clinically meaningful interpretation
	[when shown example of claims-based A&F] *"I can't explain it, thus then I would discard the graph"*	Can't explain, thus discard	A&F has no use if results are not explained	
2.3	[when shown example of claims-based A&F] *"This is comparing apples and oranges"*	Metaphor: comparing apples and oranges"	Data does not reflect clinical reality	2.3 Claims-data give an invalid representation of clinical reality
	'You will receive critic of it is not specific enough thus I can't do anything with it (…) It is caused by that stupid claims system, it is just not designed properly for this'	Not designed for this	Data is used for a purpose for which it is not suitable/valid	
2.4	*"Claims diagnoses may be inaccurate as they are registered prior to diagnostic interventions and often not adjusted after diagnostics identify an alternative diagnosis."*	Inaccurate claims data as not based on diagnostics	Numbers of intervention performed in claims data do not match numbers of interventions in reality	2.4 Claims-data give unreliable results on the number of performed interventions
	"In our own registration we have conducted a procedure a 100 times, and claims data only shows 90 procedures, then I think, where did the other 10 go?"	Anecdotal experience of wrong numbers in claims data		
	"In the past we have done a lot with it: is the practice variation the same as registration variation? And we have discovered that it just does not match 1:1 (…) that is the misery, the claims data I have come to greatly mistrust over the years, it is often not about the actual treatment that was performed"	Practice variation is not the same as registration variation<br>Greatly mistrust claims data	A&F shows only a small piece of the picture	
	"If you want to pull data from the claims system, the coding can go wrong and then you get a group out of your search which is not representative of what you actually did (…) You need to be very careful, that you think you see everything, but you are shining with a flashlight in a dark room"	Metaphor: "shining with a flashlight in a dark room"		
2.5	*"If that is the health insurer who has all that claims data, then they have an instrument to say, maybe on wrong grounds, you operate too much or you*	If health insurer has all that data<br>Wrong grounds	Health insurers can use claims-based A&F for wrong goals	2.5 Claims-based A&F may be misused by health insurers

		operate too little" (…) "or even worse, that the health insurer will say to me, you can't operate anymore, cause that is too expensive".			
		"What I fear…. is that it won't be used for the patient, but that it will be used by health insurers to condemn hospitals and that … it is tricky this"	Fear<br>Not used for the patient<br>Used to condemn hospitals<br>Tricky		
		"Yes, I greatly fear the judgement of the health insurer who is just looking over my shoulder, even though they might be right in the end that we overtreated, but yeah"	Fear of judgement by health insurers<br>Looking over my shoulder		
		"There are always fearful images of if we operate 60% and my neighbour only operates 20% of patients that the health insurer will come to me saying "you operate a little much, that is a little bit expensive, are you performing correctly?" So yeah, claims data is always a bit of a tricky thing (…) I think claims data may be a word we should avoid"	Fearful images the health insurer will say you operate too much<br>Tricky thing		
		"And let me put it this way if a health insurer says you have to do it this way or why did you do that, then I have to justify myself to the health insurer which will make the work less fun"	Health insurers says you have to do it this way<br>Justify myself		
**Conditions for implementation of claims-based A&F**	3.1	*"For wrist fractures we have long not figured out what is best for the patient, we do have ideas, but we have long not figured it out, so then the use of A&F could be dangerous"*	Have not figured out what is best for the patient<br>Dangerous	Scientific evidence on what is best treatment needs to be clear before A&F can be used	3.1 A&F only suitable for topics in which sufficient scientific evidence is available
		"Well, yeah, you should not give A&F on topics of which the truth is unknown yet, so you should not confuse the opinion of the majority with the truth"	Should not confuse opinion of the majority with the truth		
	3.2	*"I do agree with respondent (xxx) that if (the A&F) comes from our professional association or via the guideline committee, and the evidence is included in our guideline, that there will be much more support for it"*	More support if A&F comes from professional association	Distribution through professional associations gives more support for A&F	3.2 Distribution through professional association creates support among medical specialists
		"Because yes, that (the clinical guideline) is just our bible"	Clinical guideline is our bible		
		"As long as we can assume the A&F is valid, then I don't care if the insurer or the hospital distributes it"	Don't care who distributes		

3.3	*"But to fully say, here we put the division line, these hospitals are doing right and these are doing wrong based on this type of data, we should stay far away from that, I feel"*	Division line	A&F is not suitable to make judgements on "right or wrong"	3.3 Function of A&F should not be judgement of performance
	"We should use A&F, but we should not "name and shame", that is what I want to stay away from"	Name and shame		
