# Supplementary material for: Do medical specialists accept claims-based Audit and Feedback for quality improvement? A focus group study
Source: BMJ Open. 2024 Apr 8;14(4):e081063. doi: 10.1136/bmjopen-2023-081063 (PMC11015254; doi:10.1136/bmjopen-2023-081063)
Supplement: Author's manuscript [file bmjopen-2023-081063.draft_revisions.pdf]

BMJ Open is committed to open peer review. As part of this commitment we make the peer review history of every article we publish publicly available.

When an article is published we post the peer reviewers' comments and the authors' responses online. We also post the versions of the paper that were used during peer review. These are the versions that the peer review comments apply to.

The versions of the paper that follow are the versions that were submitted during the peer review process. They are not the versions of record or the final published versions. They should not be cited or distributed as the published version of this manuscript.

BMJ Open is an open access journal and the full, final, typeset and author-corrected version of record of the manuscript is available on our site with no access controls, subscription charges or pay-per-view fees (http://bmjopen.bmj.com).

If you have any questions on BMJ Open's open peer review process please email info.bmjopen@bmj.com

BMJ Open

Do medical specialists accept claims-based Audit & Feedback for quality improvement? a focus group study

Journal:	*BMJ Open*
Manuscript ID	bmjopen-2023-081063
Article Type:	Original research
Date Submitted by the Author:	18-Oct-2023
Complete List of Authors:	de Weerdt, Vera; Vrije Universiteit Amsterdam, Department of Health Economics; Amsterdam University Medical Centres<br>Ybema, Sierk; Vrije Universiteit Amsterdam, Department of Organization Sciences; Anglia Ruskin University, Department of Organization Sciences<br>Repping, S.; Amsterdam University Medical Centres<br>van der Hijden, Eric; Vrije Universiteit Amsterdam, Department of Health Economics; Zilveren Kruis Health Insurance<br>Willems, Hanna; Amsterdam University Medical Centres, Department of Geriatrics
Keywords:	Clinical audit < HEALTH SERVICES ADMINISTRATION & MANAGEMENT, Quality in health care < HEALTH SERVICES ADMINISTRATION & MANAGEMENT, MEDICAL EDUCATION & TRAINING, HEALTH SERVICES ADMINISTRATION & MANAGEMENT, Hospitals

SCHOLARONE™
Manuscripts

I, the Submitting Author has the right to grant and does grant on behalf of all authors of the Work (as defined in the below author licence), an exclusive licence and/or a non-exclusive licence for contributions from authors who are: i) UK Crown employees; ii) where BMJ has agreed a CC-BY licence shall apply, and/or iii) in accordance with the terms applicable for US Federal Government officers or employees acting as part of their official duties; on a worldwide, perpetual, irrevocable, royalty-free basis to BMJ Publishing Group Ltd ("BMJ") its licensees and where the relevant Journal is co-owned by BMJ to the co-owners of the Journal, to publish the Work in this journal and any other BMJ products and to exploit all rights, as set out in our licence.

The Submitting Author accepts and understands that any supply made under these terms is made by BMJ to the Submitting Author unless you are acting as an employee on behalf of your employer or a postgraduate student of an affiliated institution which is paying any applicable article publishing charge ("APC") for Open Access articles. Where the Submitting Author wishes to make the Work available on an Open Access basis (and intends to pay the relevant APC), the terms of reuse of such Open Access shall be governed by a Creative Commons licence – details of these licences and which Creative Commons licence will apply to this Work are set out in our licence referred to above.

Other than as permitted in any relevant BMJ Author's Self Archiving Policies, I confirm this Work has not been accepted for publication elsewhere, is not being considered for publication elsewhere and does not duplicate material already published. I confirm all authors consent to publication of this Work and authorise the granting of this licence.

For peer review only

Do medical specialists accept claims-based Audit & Feedback for quality improvement? a focus group study

V. de Weerdt[1,2], S. Ybema[3] , S. Repping[2,4], E.J.E. van der Hijden[1,5], H.C. Willems[6,7]

[1] Department of Health Economics, Faculty of Business and Economics & Talma Institute, Vrije Universiteit, Amsterdam, the Netherlands

[2] Amsterdam University Medical Centers, University of Amsterdam, Amsterdam, the Netherlands

[3] Department of Organization Sciences, Faculty of Social Sciences & Talma Institute, Vrije Universiteit, Amsterdam, the Netherlands & Anglia Ruskin University, Cambridge UK.

[4] National Healthcare Institute, Diemen, the Netherlands

[5] Zilveren Kruis Health Insurance, Zeist, the Netherlands

[6] Department of Geriatrics, Amsterdam University Medical Centers, University of Amsterdam, Amsterdam, the Netherlands

[7] Leading the Change, Zorgevaluatie Nederland, the Netherlands

Correspondence to:

V. de Weerdt

Department of Health Economics, Faculty of Business and Economics & Talma Institute, Vrije Universiteit, Amsterdam, the Netherlands, de Boelelaan 1105, 1081 HV Amsterdam, the Netherlands

(+31-6-42561363 / v.de.weerdt@vu.nl)

[version 10-06-2023]

For peer review only - http://bmjopen.bmj.com/site/about/guidelines.xhtml

Abstract

Objectives Audit & Feedback (A&F) is a widely used quality improvement (QI) intervention. However, not all feedback is accepted by professionals. While claims-based feedback has been previously used for A&F interventions, it's acceptance by professionals is unknown. This study examined medical specialists' acceptance of claims-based A&F for QI.

Design Qualitative design, with focus group discussions. Transcripts were analysed using discourse analysis.

Setting and participants A total of five online focus group discussions were conducted between May 2022 and September 2023 with 21 medical specialists from varying specialties (urology; paediatric surgery; gynaecology; vascular surgery; orthopaedics and trauma surgery) working in academic and/or regional hospitals in the Netherlands.

Results Participants described mixed views on using claims-based A&F for QI. Arguments mentioned in favour were (1) A&F stimulates reflective learning and improvement (2) claims-based A&F is more reliable than other A&F, and (3) claims-based A&F prevents administrative burden. Arguments in opposition were that (1) A&F is insufficient to create behavioural change (2) A&F lacks clinically meaningful interpretation, (3) claims data is invalid for feedback on QI, (4) claims-based A&F is unreliable, and (5) A&F may be misused by health insurers. Furthermore, participants described several conditions for implementation of A&F which shape their acceptance.

Conclusions Using claims-based A&F for QI is, for some clinical topics and under certain conditions, accepted by professionals. Acceptance of claims-based A&F can be shaped by how A&F is implemented into clinical practice. When designing A&F for QI it should be weighed whether claims data, as the most resource efficient data source, can be used or whether it is necessary to collect more specific data.

Keywords: Audit & Feedback; Clinical performance feedback; Claims data; Quality Improvement

For peer review only - http://bmjopen.bmj.com/site/about/guidelines.xhtml

STRENGTHS AND LIMITATIONS OF THIS STUDY

- Comprehensive findings due to inclusion of medical specialists from varying specialties
- Discourse analysis to create in-depth understanding of participants views on claims-based A&F
- Reflection on examples of Dutch claims-based A&F and Dutch claims data
- No inclusion of non-surgical specialties

For peer review only - http://bmjopen.bmj.com/site/about/guidelines.xhtml

Introduction

Audit and Feedback (A&F) interventions are widely used as a quality improvement (QI) strategy in healthcare (1). Through A&F, medical professionals receive feedback on their clinical performance, which intends to stimulate them to improve their quality of care (2–4). Using readily available claims data for A&F aiming to improve quality has large potential benefits, since it can prevent administrative burdens on professionals and limit costs associated with data collection for A&F (5,6).

However, a review on A&F from registries suggests that claims-based A&F may not be suitable for QI interventions, as use of claims-based A&F is associated with a lower effect on QI as opposed to A&F from quality registries (7). Healthcare claims data is collected for the purpose of reimbursement of healthcare services. This data contains patients' personal information, a diagnosis code, and billing codes for provided services and medications (8). As claims data is not collected for clinical decision making or QI, it often lacks clinical detail, information on disease severity, and relevant patient characteristics such as smoking status (5,9,10). Therefore, clinicians may not accept claims-based A&F as "fit-for-purpose" in the context of QI interventions (11,12).

Research on whether professionals accept the use of claims-based A&F in the context of quality improvement is limited. Aim of this study was to examine whether medical professionals accept claims-based A&F intended for QI purposes.

For peer review only

Method

Study Design

We conducted focus groups with medical specialists from varying specialties, to examine their potential acceptance of claims-based A&F. Focus groups were used to allow for discussions between participants on varying perceptions and arguments, thereby creating richness of results(13).

This study was designed in accordance with the COnsolidated citeria for REporting Qualitative research (COREQ) (Supplementary File 1) (14).

Contextual background of this study

In a previous study, claims-based A&F was developed in a co-creation approach with six Comparative Effectiveness Research (CER) teams (Supplementary File 2). These CER studies are conducted to identify which interventions are most effective or most cost-effective and the aim is to subsequently implement the CER results in practice to create QI. A&F in this context portrays the level in which CER results are implemented into clinical practice, which is intended to stimulate professionals to implement CER results where necessary.

In the current study, we used this previously developed claims-based A&F to explore whether independent medical specialists, i.e. who were not involved in the A&F development process, accept claims-based A&F for QI purposes.

Participants

For peer review only - http://bmjopen.bmj.com/site/about/guidelines.xhtml

For each focus group we invited medical specialists working in the specialty of the respective CER study, namely: urology, paediatric surgery, gynaecology, orthopaedics & trauma surgery, and vascular surgery. Recruitment of medical specialists was done between April 2022-June 2023 in two ways: first, we directly invited medical specialists of respective specialisms via e-mail. E-mail addresses were obtained via the CER study teams or via the professional network of the authors. Second, some of the CER study teams also contacted their professional networks via e-mail and/or newsletters.

In online focus groups, it is recommended to use a smaller number of participants to ensure active participation, thus we aimed for four – six participants per session(15). We aimed to recruit a diverse group of participants in characteristics like gender, years of work experience, geographical region, and type of hospital. We invited 93 medical specialists directly, of which 29 did not respond to the invitation and 10 refused to participate for one of the following reasons: lack of time (N=3), did not consider themselves an expert on the topic (N=5) or no reason (N=2). Of the 54 medical specialists who were interested in participation, 33 were unable to participate on the scheduled dates of the focus groups. In one of the focus groups (DART) three of the eight participants were previously familiar with each other through professional connections, in the other four focus groups none of the participants had previous relationships.

Data collection

Focus groups were held online via Microsoft Teams between April 2022 – September 2023. Focus groups were held during working hours and lasted 60-90 minutes. Researcher HW (MD-PhD) or SY (PhD) moderated the sessions. Researcher VW (MD-PhD candidate) acted as

For peer review only

For peer review only - http://bmjopen.bmj.com/site/about/guidelines.xhtml

an observer, presented the examples of claims-based A&F for CER trials and asked clarifying questions when necessary. Researchers HW and SY were not involved in the co-creation study in which the examples of claims-based A&F for CER studies were developed.

Each focus group followed the same protocol (Supplementary File 3). Focus groups consisted of two parts. First, we asked participants whether they were familiar with A&F for QI and whether they were familiar with claims-based A&F to elicit participants general perceptions on A&F and claims-based A&F. Second, we presented participants with the specific examples of claims-based A&F. These examples were visual graphs of claims-based A&F for one specific CER study, which presented the volume of the intervention studied in the CER study, per hospital in the year 2017 (Figure 1). This enabled us to capture participants' perceptions on realistic examples of claims-based A&F.

Focus groups were audio- or video-recorded and subsequently transcribed ad verbatim. Transcripts were not returned to participants.

[figure 1]

Data analysis

Transcripts were analysed using discourse analysis (16). Discourse analysis can be useful for creating an in-depth understanding of how people construct contested issues in different ways (16,17). Discourse analysis can then reveal rhetorical devices people use to make "what they are saying appear factual".

Authors VW and HW independently coded each transcript after a focus group was held, coding was done in Microsoft Word or MAXQDA. To ensure coding reliability, they discussed

For peer review only - http://bmjopen.bmj.com/site/about/guidelines.xhtml

coding of each transcript and resolved discrepancies through discussion and refined codes. In the fifth and final focus group no new codes were identified, which provided evidence for data saturation. After coding the final focus group, codes were discussed within the wider research team (VW, HW, and SY). We decided to group codes into arguments in favour and against claims-based A&F. Then, authors VW and HW re-analysed each transcript and labelled all codes as arguments in favour or against claims-based A&F and identified supporting quotes. Again, authors VW and HW discussed labels of each argument and resolved discrepancies through discussion. Subsequently, author VW grouped arguments and supporting quotes. Last, quotes were analysed using a discourse analytic approach to formulate how participants discursively presented their arguments. We did so by analysing key quotes and singling out the figures of speech and key words participants used in order to make a case for or against claims-based A&F (e.g. 'it works, because it presents a mirror'). On the basis of this first-order analysis, we then analysed what underlying discursive strategies participants adopted (e.g. presenting claims-based A&F as an effective tool for quality improvement). These strategies were discussed within the wider research team (VW, HW, SY) until all authors agreed on the formulation of the arguments.

Results

Participants

In total there were 21 participants from six clinical specialties from 17 hospitals in the Netherlands. The number of participants per focus group varied from 2-7. About half of the participants (%) were familiar with the use of A&F for QI and a minority was familiar with claims-based A&F.

For peer review only - http://bmjopen.bmj.com/site/about/guidelines.xhtml

Table 1: Participant characteristics

Focus group	Participant	Function	Type of Hospital	Gender	Familiar with A&F	Familiar with A&F based on claims data
DART	D1	Trauma surgeon	Top-clinical	M	Somewhat	No
	D2	Trauma surgeon	Top-clinical	M	Somewhat	Somewhat
	D3	Orthopaedic surgeon, traumatologist	Top-clinical	M	Somewhat	No
	D4	Trauma surgeon	Top-clinical	M	No	No
	D5	Trauma surgeon	Academic	M	Yes	No
	D6	Orthopaedic trauma surgeon	Top-clinical	M	No	Somewhat
	D7	Orthopaedic trauma surgeon	Academic	M	No	No
STONE	S1	Urologist	Top-clinical	F	Yes	Yes
	S2	Urologist	Top-clinical	M	Yes	Yes
	S3	Urologist	Academic	M	Yes	Yes
	S4	Urologist	General	F	No	No
Proclion	P1	Vascular surgeon	Top-clinical	M	Somewhat	Somewhat
	P2	Vascular surgeon	Top-clinical	M	Yes	No
MIRA2	M1	Gynaecologist	Generic	F	Yes	Yes
	M2	Gynaecologist	Top-clinical	F	Yes	Yes
	M3	Gynaecologist	Academic	M	Yes	Yes
	M4	Gynaecologist	Academic	F	Yes	Yes
	M5	Gynaecologist	Generic	F	Yes	Yes
CAPP	C1	Paediatric surgeon	Academic	M	Yes	No
	C2	Paediatric surgeon	Top-clinical	F	Yes	No
	C3	Paediatric surgeon	Top-clinical	M	Yes	No

Acceptance of claims-based Audit & Feedback

Most participants were positive in their views on A&F in general, but participants were mixed in their views on claims-based A&F and the specific examples of claims-based A&F for CER studies. While some participants were unambiguously in favour or against claims-based A&F, most participants posed arguments for both sides (Table 1). Participants who were familiar with A&F were more positive towards claims-based A&F, than participants who were unfamiliar with A&F. Furthermore, participants who were in favour of A&F for QI, argued their stance depended on how claims-based A&F was implemented in practice.

For peer review only - http://bmjopen.bmj.com/site/about/guidelines.xhtml

1. Arguments in favour of claims-based A&F

1.1. A&F stimulates reflective learning and improvement

First, participants who viewed A&F favourably described it as a tool that creates reflective learning and improvement. They believed A&F could contribute to QI by providing feedback on whether CER results are implemented into clinical practice. Participants described the presented visual graphs of claims-based A&F as a "*visually attractive tool"* to initiate change. They believed feedback could stimulate a conversation on change. To explain their positive assessment, they used metaphors to frame A&F as a tool that activates and accelerates change: "*A&F is setting everything in motion"*, and as an instrument that stimulates self-critical reflection: "*Showing people a mirror is why it works*", because, as another participant put it: *"By comparing yourself you gain insights into whether you are doing "the right thing"".* Some participants describe they believe A&F is mostly useful for outliers. The imagery some participants used reflected their positive reception of A&F as a tool for change.

1.2 Claims-based A&F is more reliable than other types of A&F

A second argument in favour of claims-based A&F was that it is more reliable than other types of A&F, as claims-data is perceived as more reliable than self-reported data. Participants describe:

> *"If you can get it out of claims data, then you have objective data on your own process, cause otherwise it is dependent on what the person registers and that can be less reliable"*

For peer review only

For peer review only - http://bmjopen.bmj.com/site/about/guidelines.xhtml

"now it is depended on the administrator whether A&F is accurate, if you can enhance that through A&F based on data of other sources, than you have a better and more accurate registration which gives better A&F"

Here, participants describe self-reported data negatively by labelling it as potentially "less reliable" or "inaccurate", while they describe claims data as "objective" and "more accurate". These participants pose that "objective" and "more accurate" claims data, leads to "better A&F".

1.3 Claims-based A&F prevents administrative burden on professionals

Another argument which participants valued was that use of claims data as the source for A&F interventions prevents administrative burden on professionals. Participants describe: "*I think it is very valuable information, if we can collect it easily without additional (administrative) burden".* The key word "if" shows that the perceived valuableness of A&F is conditional upon the administrative burden the collection of A&F puts on professionals. Several participants describe the readily availability of claims data as an advantage for A&F.

1. Arguments against claims-based A&F

1.1. A&F is insufficient to create change of clinical behaviour

A first counterargument to use A&F was that it is insufficient to create change of clinical behaviour. Some participants question the effectiveness of A&F:

"I wonder then if you would have the same data and would show it to hospitals

For peer review only

if that would really lead to change of practice. I don't know yet. Maybe they will still think of explanations for why they deviate from the norm"

Another participant describes the insufficiency of A&F as:

"If you get the A&F, you need to act upon it, but you can't reinforce that, so… there is the dilemma: you can show it, but subsequently you want change, but that change depends on individual hospitals, so…".

Keywords are "dilemma" and "reinforce", the speaker hereby assumes that for behavioural change to happen in professionals this must be enforced, which A&F itself does not do. Another participant described that A&F for him does not create the intended QI, but it only incentives him to register claims differently:

"if you have the feeling you are doing evidence-based treatments and that all it takes is to register claims codes differently, then you will change claims codes:, we are not more catholic than the pope"

Here, the participant used the metaphor "we are not more catholic than the pope" to justify that he does not change his clinical behaviour, but only registers claim codes differently, hereby posing that changing clinical behaviour is more strict than necessary. Thus, participants describe the insufficiency of A&F to create QI, by questioning its effectiveness or by contriving explanations for why A&F will not lead to QI.

1.2. Claims-based A&F lacks clinically meaningful interpretation

A second argument against A&F was that participants felt it was not possible to interpret the visual graphs of claims-based A&F in a clinically meaningful way. Participants questioned the benefits of the data: *"Yes, it is a nice overview. Only, ehm yes, what are*

For peer review only - http://bmjopen.bmj.com/site/about/guidelines.xhtml

the consequences of this figures? I can't .. ehm.. I can't see how to translate this to my practice? [...] How do we or the patient benefit from this exactly?". Furthermore, some participants stated more strongly that if they could not interpret the data they would not use it: "*I can't explain these results, thus then I would discard the feedback*".

2.3 Claims data gives an invalid representation of clinical reality

The third counterargument was that claims data gives an invalid representation of clinical reality. When presented with the visual graphs of claims-based A&F on CER, for three out of five cases the participants described the visual graphs negatively: "*It is comparing apples and oranges*" or *"You will receive critic of it is not specific enough thus I can't do anything with it (…) It is caused by that stupid claims system, it is just not designed properly for this"*. Using the metaphor "comparing apples and oranges" shows the participant believes an unjust comparison is made, in this case the claims-based A&F which is not a valid comparison with the CER population. Furthermore, "*not designed … for this*" also describes that the participant believes claims data is used for a purpose (A&F on CER), for which it is not suitable or valid. Use of the word "stupid" also shows the participants' frustration with the claims system. Thus, for three out of five cases, the participants describe the claims-based A&F as invalid feedback on CER.

1.3. Claims data gives unreliable results on the number of performed interventions

A fourth argument against claims-based A&F was that claims data gives unreliable results on the number of performed interventions, thus claims-based A&F is not reliable either.

For peer review only - http://bmjopen.bmj.com/site/about/guidelines.xhtml

Participants described personal experiences with registration of claims data and with claims-based A&F to illustrate their point:

> *"Claims diagnoses may be inaccurate as they are registered prior to diagnostic interventions and often not adjusted after diagnostics identify an alternative diagnosis".*
>
> *"In our own registration we have conducted a procedure a 100 times and claims data only shows 90 procedures, then I think where did the other 10 go?"*

Participants emphasized their mistrust of claims data by stating:

> *"Over the years I have started to greatly mistrust claims data"*
>
> *"If you want to pull data from the claims system, the coding can go wrong and then you get a group out of your search which is not representative of what you actually did (…) you need to be very careful with this: you may think you see everything, but you're only using a flashlight in a dark room."*

Here participants use the word "greatly" to emphasize their mistrust in claims data and the metaphor "only using a flashlight in a dark room" to illustrate their perception that claims-based A&F can only show a small part of clinical practice, which may lead to incorrect judgements about clinical performance.

Thus, the participants use anecdotal experiences to convey objectiveness in their arguments against claims-based A&F and figures of speech to emphasize their objections of claims-based A&F.

For peer review only - http://bmjopen.bmj.com/site/about/guidelines.xhtml

2.5 Claims-based A&F may be misused by health insurers

The last argument against claims-based A&F was that participants feared the role of the health insurer. Participants described that they feel health insurers may have a conflict of interest, such as financial incentives to decrease volumes of care, instead of QI purposes. "*If that is the health insurer who has all that claims data, then they have an instrument to say, maybe on wrong grounds, you operate too much or you operate too little*" and "*or even worse, that the health insurer will say to me, you can't operate anymore, cause that is too expensive*".

Keywords here are "*wrong grounds*" in which the speaker assumes that health insurers make decisions based on wrong grounds and "*even worse*" to emphasize the severity of their argument.

Furthermore, participants describe that they fear the role of health insurers when using claims-based A&F:

> *"What I fear…. is that it won't be used for the patient, but that it will be used by health insurers to condemn hospitals and that …it is tricky this"*

> *"Yes, I greatly fear the judgement of the health insurer who is just looking over my shoulder, even though they might be right in the end that we overtreated, but yeah"*

> *"There are always fearful images of if we operate 60% and my neighbor only operates 20% of patients that the health insurer will come to me saying "you operate a little much, that is a little bit expensive, are you performing correctly?" So yeah, claims data is always a bit of a tricky thing (…) I think claims data may be a thing we should avoid"*

Commented [WVd(1]: fearful

For peer review only

For peer review only - http://bmjopen.bmj.com/site/about/guidelines.xhtml

Here, the participants legitimize their objections to claims-based A&F by posing that the role of health insurers is something that should be "feared".

Furthermore, participants describe that they do not want to justify their behaviour to the health insurer, as this leads to less joy in work:

> *"And if a health insurer says, (…) you have to do it this way or why did you do that, then I have to justify myself to the health insurer, which will make the work less fun"*

Thus, the participants use several types of discourse to describe the role of health insurers in claims-based A&F as negative.

1. Implementation of claims-based A&F in practice

1.1. A&F only suitable for topics in which sufficient clinical evidence is available

Participants argue that A&F is only suitable for topics on which scientific evidence is available for what is considered "best practice". To emphasize their argument, they describe using A&F without scientific evidence as "dangerous":

> *"for wrist fractures we have long not figured out what is best for the patient, we do have ideas, but we have long not figured it out, so then the use of A&F could be dangerous"*

Another participant describes:

> *"Well, yeah, you should not give A&F on topics of which the truth is unknown yet, so you should not confuse the opinion of the majority with the truth"*

This participant describes scientific evidence as "*the truth*" and poses that A&F on topics without scientific evidence may cause bias in misconceiving the clinical practice of the majority is "*the best*".

For peer review only - http://bmjopen.bmj.com/site/about/guidelines.xhtml

3.2 Distribution through professional association creates support among medical specialists

Furthermore, participants describe that distribution of A&F done via their professional associations, instead of health insurers, is more likely to be accepted:

> *"I do agree that if A&F comes through our professional association or guideline committee and it is included in our own guideline that there will just be much more support for it"*

A participant emphasizes this point by stating:

> *"because yes, that (the clinical guideline) is just our bible"*

The participant uses the word "bible" to emphasize the value of and support for clinical guidelines of professional associations.

However, other participants state that who distributes the data is unimportant to them:

> *"as long as we can assume the A&F is valid, then I don't care if the insurer or the hospital distributes it"*

3.3 Function of A&F should not be judgement of performance

Lastly, participants argued claims-based A&F should not be used to judge whether clinical performance is "wrong" or "right":

> *"but to fully say, here we put the division line, these hospitals are doing right and these are doing wrong based on this type of data, we should stay far away from that, I feel"*

For peer review only

"We should use A&F, but we should not "name and shame", that is what I want to stay away from"

Here participants' use of the words "division line" and "name and shame" show their negative perceptions on judgement of performance based on A&F.

For peer review only

Table 1. Results discourse analysis

		Quotes	Keywords or Figures of speech	Discourse	Argument
In favor of claims-based A&F	1.1	*"I think this (claims-based A&F) is a visual attractive thing to set everything in motion (…) and to stimulate implementation of result results, I do think it helps"*	Sets in motion	A&F gives insight in own performance and thereby creates action	1.1 A&F stimulates reflective learning and improvement
		"Presenting a mirror is why it works"	Comparison of A&F to a mirror		
		"A&F is useful to start a conversation for change, A&F is the first step to initiate change"	Start<br>First step		
	1.2	*"Claims data can give a more reliable portrayal of clinical practice, thus then you get reliable A&F: now it is dependent on the administrator whether A&F is accurate, if you can enhance that through A&F based on data of other sources, then you have a better and more accurate registration which gives better A&F"*	Reliable<br>Accurate	Claims-data is more reliable than self-reported data, thus claims-based A&F is more reliable than other types of A&F	1.2 Claims-based A&F is more reliable than other types of A&F
		"If you can get it out of claims data, then you have objective data on your own process, because otherwise it is dependent on what the person registers and that can be less reliable"	Objective data		
	1.3	*"I think it is very valuable information, if we can collect it easily without additional (administrative) burden"*	If (conditionality)<br>Collect easily	Valuableness of claims-based A&F is conditional upon administrative burden for professionals	1.3 Claims-based A&F prevents administrative burden
Op po si[illegible]	2.1	*"I wonder then if you would have the same data and would show it to hospitals if that would really lead to change of practice. I don't know yet. Maybe they will still think of reasons to deviate from the norm"*	Wonder if that would lead to change	Questioning the effect of claims-based A&F	2.1 Claims-based A&F is insufficient to create change of clinical behavior
		"If you get the A&F, you need to act upon it, but you can't reinforce that, so… there is the dilemma: you can show it, but subsequently you want change, but that change depends on individual hospitals, so…".	Can't reinforce (…) action<br>Dilemma	Questioning the effect of claims-based A&F	
		"if you have the feeling you are doing evidence-based treatments and that all it takes is to register claim codes differently, then you will change claims code,	Change claims code	Posing claims-based A&F leads to different	

For peer review only

		we are not more catholic than the pope”	Metaphor: not more catholic than the pope	registration of claims-codes, not change of clinical behavior	
	2.2	[when shown example of claims-based A&F] *“I can’t explain it, thus then I would discard the graph”*	Can’t explain, thus discard	A&F has no use if results are not explained	2.2 Claims-based A&F lacks clinically meaningful interpretation
		[when shown example of claims-based A&F] *“Yes, it is a nice overview. Only, ehm yes, what are the consequences of this figures? I can’t .. ehm.. I can’t see how to translate this to my practice? [...] How do we or the patient benefit from this exactly?”.*	How do we or the patient benefit?	Questioning the relevance of the A&F	
	2.3	*“You need to be very careful, that you think you see everything, but you are shining with a flashlight in a dark room”*	Metaphor: “shining with a flashlight in a dark room”	A&F shows only a small piece of the picture	2.3 Claims-data gives an invalid representation of clinical reality
		[when shown example of claims-based A&F] *“This is comparing apples and oranges”*	Metaphor: comparing apples and oranges”	Data does not reflect clinical reality	
	2.4	*“In the past we have done a lot with it: is the practice variation the same as registration variation? And we have discovered that it just does not match 1:1 (…) that is the misery, the claims data I have come to greatly mistrust over the years, it is often not about the actual treatment that was performed”*	Practice variation is not the same as registration variation<br>Greatly mistrust claims data	Numbers of intervention performed in claims data do not match numbers of interventions in reality	2.4 Claims-data gives unreliable results on number of interventions performed
		“in our own registration I performed a procedure 100 times, in claims data I only see 90, then I think, where did the other 10 go?”	Anecdotal experience<br>Wrong numbers in claims data		
	2.5	*“If that is the health insurer who has all that claims data, then they have an instrument to say, maybe on wrong grounds, you operate too much or you operate too little” (…) “or even worse, that the health insurer will say to me, you can’t operate anymore, cause that is too expensive”.*	If health insurer has all that data<br>Wrong grounds	Health insurers can use claims-based A&F for wrong goals	2.5 Claims-based A&F may be misused by health insurers
		“What I fear…. is that it won’t be used for the patient, but that it will be used by health insurers to condemn hospitals and that … tricky this”	Fear<br>Not used for the patient<br>Used to condemn hospitals<br>Tricky		
A &F			Fear judgement of health insurer		

For peer review only

		"Yes, I greatly fear the judgement of the health insurer who is just looking over my shoulder, even though they might be right in the end that we overtreated, but yeah"	Looking over my shoulder		
		"There are always fearful images of if we operate 60% and my neighbor only operates 20% of patients that the health insurer will come to me saying "you operate a little much, that is is little bit expensive, are you performing correctly?" So yeah claims data is always a bit of a tricky word (…) I think claims data may be a word we should avoid"	Fearful images the health insurer will say you operate too much<br>Tricky this		
		"Let me put it this way if a big health insurer says you have to do it this way of why did you do that, then I have to justify myself to the health insurer which will make the work less fun"	Health insurers says you have to do it this way<br>Justify myself		
**Conditions for implementation of claims-based A&F**	3.1	*"For wrist fractures we have long not figured out what is best for the patient, we do have ideas, but we have long not figured it out, so then the use of A&F could be dangerous"*	Have not figured out what is best for the patient<br>Dangerous	Scientific evidence on what is best treatment needs to be clear before A&F can be used	3.1 A&F can only be used if scientific evidence exists for best practice
		"Well, yeah, you should not give A&F on topics of which the truth is unknown yet, so you should not confuse the opinion of the majority with the truth"	Should not confuse opinion of the majority with the truth		
	3.2	*"I do agree with respondent (xxx) that if (the A&F) comes from our professional society or via the guideline committee, and the evidence is included in our guideline, that that gives much more support because yes, that (the clinical guideline) is just our bible"*	More support if A&F comes from professional society<br>Clinical guideline is our bible	Distribution through professional society gives more support for A&F	3.2 Distribution through professional society gives more support for A&F
		"Distribution through professional society gives more support for A&F"	Don't care who distributes		
	3.3	*"But to fully say, here we put the division line, these hospitals are doing right and these are doing wrong based on this type of data, we should stay far away from that, I feel"*	Division line	A&F is not suitable to make judgements on "right or wrong"	3.3 A&F should not be used for judgement
		"We should use A&F, but we should not "name and shame", that is what I want to stay away from"	Name and shame		

For peer review only

Discussion

This study examined medical specialists' acceptance of claims-based A&F for QI through focus group discussions, in which participants were presented with examples of claims-based A&F on CER trials. While participants were mostly positive in their views on A&F in general, they were mixed in their views on claims-based A&F and on the specific examples of claims-based A&F for CER trials. Most participants put forward arguments both in favour and against claims-based A&F. Arguments in **favour** of claims-based A&F were that (1) A&F stimulates reflective learning and improvement (2) claims-based A&F is more reliable than other A&F (3) claims-based A&F prevents administrative burden. The **opposition** against A&F as a QI tool was articulated in five ways: participants claimed that (1) A&F is insufficient to create change of clinical behaviour; (2) A&F lacks clinically meaningful interpretation; (3); claims data is perceived as invalid for feedback on QI; (4) claims-based A&F is perceived as unreliable and (5) claims-based A&F may be misused by health insurers. Additionally, participants indicated that their acceptance depended on how claims-based A&F would be implemented: (1) there should be sufficient clinical evidence for the topic of A&F; (2) A&F should be distributed by professional societies; and (3) A&F should not be used to impose judgment on clinical performance.

Participants had varying views on the effectiveness of A&F as a tool to create QI. We observed that participants without prior use of A&F were more sceptical of A&F as a tool than participants who had used A&F prior to the focus group. In line with previous research, this suggests being unknown with A&F, makes it unloved. While A&F has been used as QI intervention in healthcare for years, studies consistently report physicians' incapability to

For peer review only - http://bmjopen.bmj.com/site/about/guidelines.xhtml

interpret population-level feedback (18–21). This leads to negative perceptions regarding the usefulness of A&F, which decreases acceptance of A&F(19). Thus, our findings seem to corroborate earlier findings that physicians should be trained on how to interpret and use A&F for QI (19,20). However, our findings also suggest that acceptance of A&F does not depend solely on informing and training physicians.

Data validity and reliability was mentioned by participants as the most important factor to accept claims-based A&F for QI purposes. Again, the participants gave mixed reactions, some viewed claims data as potentially more reliable than self-reported data, while others questioned the reliability of claims data and the validity of claims-based A&F for QI. Studies examining acceptance of A&F consistently report data quality as the main factor for accepting A&F, irrespective of which data source is used for the feedback (3,20–24). Other studies on claims-based A&F found physicians had mainly negative perceptions on the quality of claims data(11,20,21), while we also find positive views. For claims-based A&F, it is important to note that the validity and reliability are dependent on the clinical topic for which A&F is used. For example, claims data captures whether a patient had a wrist fracture, but lacks detail of the location of the fracture. This detail may not be relevant for reimbursement, but can be clinically relevant in the context of QI, thereby decreasing the "fit-for-purpose" of using claims data for A&F for QI. The validity or "fit-for-purpose" for A&F on QI is determined by how specific claims data distinguishes clinically relevant patient groups. For some healthcare fields, claims data is more detailed than others, increasing the "fit-for-purpose" of claims-based A&F for QI within these specialties(25). The reliability of claims data may also vary for clinical topics: participants in our study noted that claims data is registered more reliable for larger procedures (e.g., surgeries) as compared to smaller

For peer review only - http://bmjopen.bmj.com/site/about/guidelines.xhtml

interventions and diagnoses for acute, conservatively treated disorders. Thus, the applicability of claims data for A&F in QI is contingent on the clinical topic.

While lack of data quality is consistently mentioned as a barrier for using A&F, it is important to note that these objections are not only about the data itself, but professionals' perceptions play a big role in accepting the data as accurate or not. For example, data accuracy is questioned more strongly when A&F shows performance is low or when performance is judged(3,23). Data accuracy may also be less questioned in A&F interventions that are voluntary and/or physician-led (19,26).

Participants in our study also suggested to distribute claims-based A&F via professional associations, instead of via health insurers. This is in line with other research, which finds A&F is more accepted and effective if it comes from a colleague instead of an external source(1,3). While endorsement of A&F by professional associations could be beneficial in its acceptance by physicians, studies suggest that A&F is most effective if it is given by a direct colleague (1,27). Thus, it would be interesting to examine whether endorsement of A&F by professional associations is sufficient to create QI or whether local champions of A&F are necessary to create change.

In our study we only examined medical specialists' acceptance of the data source used for A&F: claims data. However, acceptance of claims data as the source for A&F does not guarantee medical specialists' acceptance of an A&F intervention. This is also dependent on the content of the feedback (e.g. is it timely, personalized); recipient characteristics (e.g. do they have an internal/external locus of control) and the audit method (e.g. was it transparent) (3,20,22,28). One study posed acceptance of A&F is unnecessary and that the goal of changing clinical behaviour can also be achieved by combining A&F with for example

For peer review only - http://bmjopen.bmj.com/site/about/guidelines.xhtml

financial incentives (29). However, lack of physicians' acceptance of A&F will likely not create a sustainable QI culture among physicians.

Our study suggests that, under certain conditions, medical specialists can accept claims-based A&F for QI purposes. In the review of van de Veer et al, it is uncertain whether the source of A&F being claims data is the explaining factor for lower effectiveness of the interventions. Even if claims data is not the most effective data source, it is currently the most resource efficient data source for A&F interventions, especially for large-scale interventions aiming to provide A&F to multiple providers. Thus, policymakers, managers and professionals should weigh whether the clinical topic can be portrayed by claims-based A&F or whether it is necessary to collect more detailed data for A&F aiming to improve quality.

Strengths & weaknesses

This study examined medical specialists' acceptance of claims-based A&F in the context of QI. Through discourse analysis we were able to create a thorough understanding in what factors influence medical specialists' acceptance of claims-based A&F. Strength of this study is that we used realistic examples of claims-based A&F on varying CER studies and included medical specialists from various clinical specialities. Another strength of this study if that we included both participants who were and were not familiar with A&F and claims-based A&F, which increases the generalizability of our results.

A limitation of this study is that participants in our study reflected on Dutch claims data. As the content of claims data and the accuracy of registration can vary by country, medical specialists in other countries may view claims-based A&F differently. However, our results describe themes which are common across healthcare systems and our results corroborate

For peer review only - http://bmjopen.bmj.com/site/about/guidelines.xhtml

and detail previous findings described in A&F literature. Secondly, the participants in our focus groups were mainly surgeons, thus it is unclear whether other healthcare professionals put forward additional perspectives on claims-based A&F. Lastly, in some focus groups we had a limited number of participants which may have decreased the level of discussion between participants. However, we did reach data saturation over all focus groups, thus we do not believe that additional arguments would have been identified with more participants per focus group.

Conclusion

Using claims-based A&F for quality improvement is, for some clinical topics and under certain conditions, accepted by medical specialists. Perceptions of medical specialists on A&F in general and claims-based A&F for QI varied, and acceptance of claims-based A&F can be shaped by how A&F is implemented into clinical practice. Currently, claims data is the most resource efficient data source for A&F interventions. Thus, policymakers, managers and professionals should weigh whether the clinical topic can be portrayed by claims-based A&F or whether it is necessary to collect other data for A&F aiming to improve quality.

For peer review only

References

1. Ivers N, Jamtvedt G, Flottorp S, Young JM, Odgaard-Jensen J, French SD, et al. Audit and feedback: Effects on professional practice and healthcare outcomes. Cochrane Database of Systematic Reviews. 2012;2012(6).
2. Gude WT, Van Engen-Verheul MM, Van Der Veer SN, De Keizer NF, Peek N. How does audit and feedback influence intentions of health professionals to improve practice? A laboratory experiment and field study in cardiac rehabilitation. BMJ Qual Saf. 2017;26(4):279–87.
3. Brown B, Gude WT, Blakeman T, Van Der Veer SN, Ivers N, Francis JJ, et al. Clinical Performance Feedback Intervention Theory (CP-FIT): A new theory for designing, implementing, and evaluating feedback in health care based on a systematic review and meta-synthesis of qualitative research. Implementation Science. 2019;14(1):1–25.
4. Gude WT, Brown B, Van Der Veer SN, Colquhoun HL, Ivers NM, Brehaut JC, et al. Clinical performance comparators in audit and feedback: A review of theory and evidence. Implementation Science. 2019;14(1):1–14.
5. Mckee M. Routine data: A resource for clinical audit ? Quality in Health Care. 1994;2(2):104–11.
6. Solberg LI, Engebretson KI, Sperl-Hillen JM, Hroscikoski MC, O'Connor PJ. Are claims data accurate enough to identify patients for performance measures or quality improvement? The case of diabetes, heart disease, and depression. American Journal of Medical Quality. 2006;21(4):238–45.
7. van der Veer SN, de Keizer NF, Ravelli ACJ, Tenkink S, Jager KJ. Improving quality of care. A systematic review on how medical registries provide information feedback to health care providers. Int J Med Inform [Internet]. 2010;79(5):305–23. Available from: http://dx.doi.org/10.1016/j.ijmedinf.2010.01.011
8. Peek N, Holmes JH, Sun J. Technical challenges for big data in biomedicine and health: data sources, infrastructure, and analytics. Yearb Med Inform. 2014;9:42–7.
9. Powell AE, Davies HTO, Thomson RG. Using routine comparative data to assess the quality of health care: Understanding and avoiding common pitfalls. Qual Saf Health Care. 2003;12(2):122–8.
10. Iezzoni LI. Annals of Internal Medicine Assessing Quality Using Administrative Data Content of Administrative Databases. Ann Intern Med. 1997;127(2):666–74.
11. van Hoof Thomas; Pearson, David; Giannotti, Tierney; Tate, Janet; Elwell, Anne; Barr JM. Lessons Learned from Performance Feedback. Journal for Healthcare Quality. 2001;28(3):20–31.
12. Bonis PAL. Quality incentive payment systems: Promise and problems. J Clin Gastroenterol. 2005;39(4 SUPPL.):176–82.
13. Freitas H, Oliveira M, Jenkins M, Popjoy O. The Focus Group, A Qualitative Research Method. Isrc. 1998;(010298):1–22.
14. Tong A, Sainsbury P, Craig J. Consolidated criteria for reporting qualitative research (COREQ): A 32-item checklist for interviews and focus groups. International Journal for Quality in Health Care. 2007 Dec;19(6):349–57.
15. Lobe B. A new era in focus group research: challenges innovation and practice. Barbour R, Morgan D, editors. London: Palgrave Macmillan; 2017. 1–14 p.
16. Cheek J. At the margins? Discourse analysis and qualitative research. Qual Health Res. 2004;14(8):1140–50.

For peer review only - http://bmjopen.bmj.com/site/about/guidelines.xhtml

17. Stevens P, Harper DJ. Professional accounts of electroconvulsive therapy: A discourse analysis. Soc Sci Med. 2007;64(7):1475–86.

18. N., Ivers, J., Barnsley, R., Upshur, K., Tu, B., Shah, J. G. My approach to this job is. One person at a time: Perceived discordance between population-Level quality targets and patient-Centred care. Canadian Family Physician [Internet]. 2014;60(3):258–66. Available from: http://www.cfp.ca/content/60/3/258.full.pdf+html%5Cnhttp://ovidsp.ovid.com/ovidweb.cgi?T=JS&PAGE=reference&D=emed12&NEWS=N&AN=2014204124

19. Desveaux L, Ivers NM, Devotta K, Ramji N, Weyman K, Kiran T. Unpacking the intention to action gap: a qualitative study understanding how physicians engage with audit and feedback. Implementation Science. 2021;16(1):1–9.

20. Van Braak M, Visser M, Holtrop M, Statius Muller I, Bont J, Van Dijk N. What motivates general practitioners to change practice behaviour? A qualitative study of audit and feedback group sessions in Dutch general practice. BMJ Open. 2019;9(5):1–8.

21. De Bekker PJGM, De Weerdt V, Vink MDH, Van Der Kolk AB, Donker MH, Van Der Hijden EJE. "Give me something meaningful": GPs perspectives on how to improve an audit and feedback report provided by health insurers - an exploratory qualitative study. BMJ Open Qual. 2022;11(4):1–9.

22. Payne VL, Hysong SJ. Model depicting aspects of audit and feedback that impact physicians' acceptance of clinical performance feedback. BMC Health Serv Res [Internet]. 2016;16(1):1–12. Available from: http://dx.doi.org/10.1186/s12913-016-1486-3

23. Hysong SJ, Smitham K, SoRelle R, Amspoker A, Hughes AM, Haidet P. Mental models of audit and feedback in primary care settings. Implementation Science. 2018;13(1):1–11.

24. Rouleau G, Reis C, Ivers N, Desveaux L. Characterizing the Gaps Between Best-Practice Implementation Strategies and Real-world Implementation: Qualitative Study Among Family Physicians Who Engaged With Audit and Feedback Reports. JMIR Hum Factors. 2023;10:1–12.

25. Ulyte A, Bähler C, Schwenkglenks M, Von Wyl V, Gruebner O, Wei W, et al. Measuring diabetes guideline adherence with claims data: Systematic construction of indicators and related challenges. BMJ Open. 2019;9(4):1–8.

26. Hysong SJ, Best RG, Pugh JA. Audit and feedback and clinical practice guideline adherence: Making feedback actionable. Implementation Science. 2006;1(1):1–10.

27. Hysong SJ. Meta-Analysis: Audit and feedback features impact effectiveness on care quality. Med Care. 2009;47(3):356–63.

28. Yi SG, Wray NP, Jones SL, Bass BL, Nishioka J, Brann S, et al. Surgeon-specific performance reports in general surgery: An observational study of initial implementation and adoption. J Am Coll Surg [Internet]. 2013;217(4):636-647.e1. Available from: http://dx.doi.org/10.1016/j.jamcollsurg.2013.04.040

29. Ilgen DR, Fisher CD, Taylor MS. Consequences of individual feedback on behaviour in organizations. Journal of Applied Psychology. 1979;64(4):349–71.

For peer review only - http://bmjopen.bmj.com/site/about/guidelines.xhtml

For peer review only

Declarations

Ethics approval and consent to participate

This research was conducted in accordance with the ethical guidelines of the Research Ethics Review Committee (BETHCIE) of the Faculty of Science, Vrije Universiteit. Based on a self-assessment, the BETHCIE research committee approved the research protocol. All medical specialists and provided informed consent for participation in this study and publication of results.

Competing interests

The authors declare that they have no competing interests.

Funding

This research was carried out within the Academic Working Place Care Practice and Policy of the Consortium Quality of Care of the Netherlands Federation of University Medical Centers (NFU), and National Health Care Institute.’ This funder had no role in the design of the study, data collection, data analysis, interpretation of data and writing the manuscript.

For peer review only - http://bmjopen.bmj.com/site/about/guidelines.xhtml

Authors' contributions

Researchers HW, VW and EvdH designed the study. Researcher SY was involved in the study for his extensive expertise in qualitative research and discourse analysis. Researcher HW and/or SY moderated the focus groups, researcher VW was present in the focus groups as an observer and presenter. All researchers were involved in the write-up of this study.

For peer review only

For peer review only - http://bmjopen.bmj.com/site/about/guidelines.xhtml

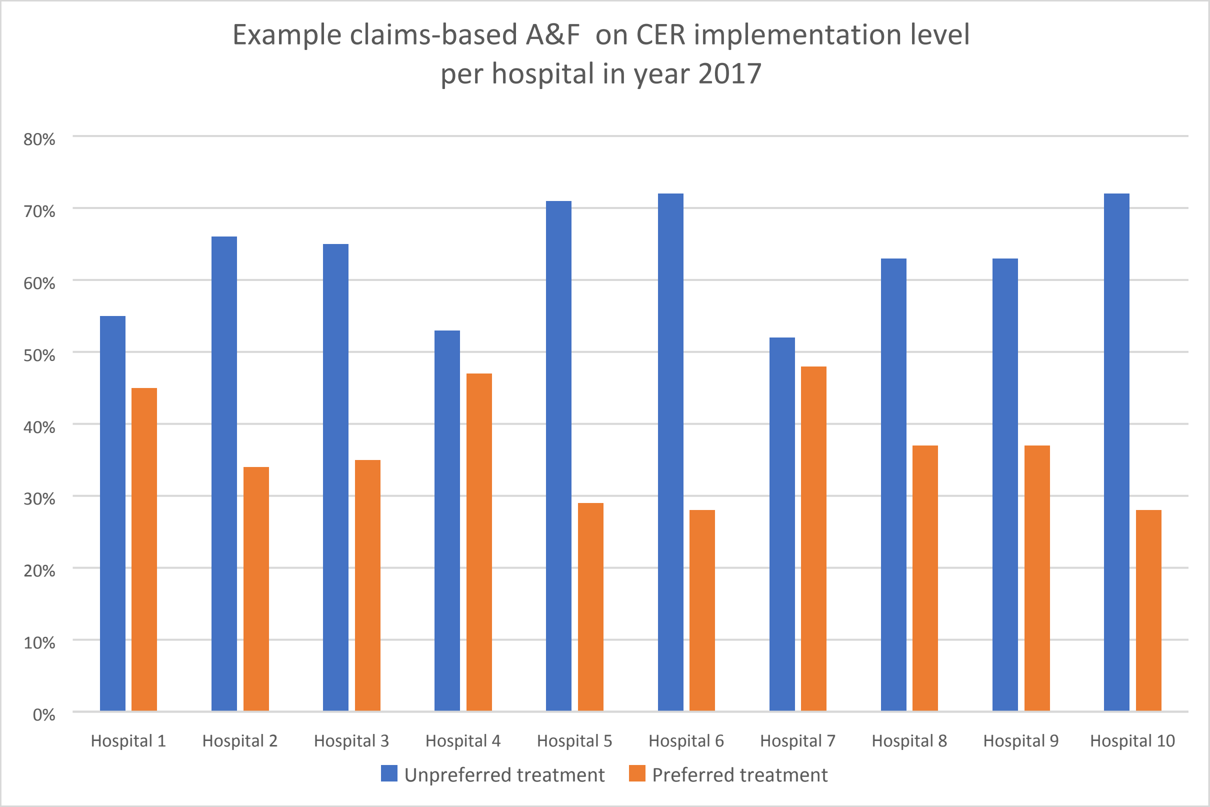

Figure 1: Example of claims-based A&F on CER implementation level per hospital

COREQ (COnsolidated criteria for REporting Qualitative research) Checklist

A checklist of items that should be included in reports of qualitative research. You must report the page number in your manuscript where you consider each of the items listed in this checklist. If you have not included this information, either revise your manuscript accordingly before submitting or note N/A.

Topic	Item No.	Guide Questions/Description	Reported on Page No.
**Domain 1: Research team and reflexivity**			
Personal characteristics			
Interviewer/facilitator	1	Which author/s conducted the interview or focus group?	
Credentials	2	What were the researcher's credentials? E.g. PhD, MD	
Occupation	3	What was their occupation at the time of the study?	
Gender	4	Was the researcher male or female?	
Experience and training	5	What experience or training did the researcher have?	
Relationship with participants			
Relationship established	6	Was a relationship established prior to study commencement?	
Participant knowledge of the interviewer	7	What did the participants know about the researcher? e.g. personal goals, reasons for doing the research	
Interviewer characteristics	8	What characteristics were reported about the inter viewer/facilitator? e.g. Bias, assumptions, reasons and interests in the research topic	
**Domain 2: Study design**			
Theoretical framework			
Methodological orientation and Theory	9	What methodological orientation was stated to underpin the study? e.g. grounded theory, discourse analysis, ethnography, phenomenology, content analysis	
Participant selection			
Sampling	10	How were participants selected? e.g. purposive, convenience, consecutive, snowball	
Method of approach	11	How were participants approached? e.g. face-to-face, telephone, mail, email	
Sample size	12	How many participants were in the study?	
Non-participation	13	How many people refused to participate or dropped out? Reasons?	
Setting			
Setting of data collection	14	Where was the data collected? e.g. home, clinic, workplace	
Presence of non-participants	15	Was anyone else present besides the participants and researchers?	
Description of sample	16	What are the important characteristics of the sample? e.g. demographic data, date	
Data collection			
Interview guide	17	Were questions, prompts, guides provided by the authors? Was it pilot tested?	
Repeat interviews	18	Were repeat inter views carried out? If yes, how many?	
Audio/visual recording	19	Did the research use audio or visual recording to collect the data?	
Field notes	20	Were field notes made during and/or after the inter view or focus group?	
Duration	21	What was the duration of the inter views or focus group?	
Data saturation	22	Was data saturation discussed?	
Transcripts returned	23	Were transcripts returned to participants for comment and/or	

For peer review only

Topic	Item No.	Guide Questions/Description	Reported on Page No.
		correction?	
**Domain 3: analysis and findings**			
Data analysis			
Number of data coders	24	How many data coders coded the data?	
Description of the coding tree	25	Did authors provide a description of the coding tree?	
Derivation of themes	26	Were themes identified in advance or derived from the data?	
Software	27	What software, if applicable, was used to manage the data?	
Participant checking	28	Did participants provide feedback on the findings?	
Reporting			
Quotations presented	29	Were participant quotations presented to illustrate the themes/findings? Was each quotation identified? e.g. participant number	
Data and findings consistent	30	Was there consistency between the data presented and the findings?	
Clarity of major themes	31	Were major themes clearly presented in the findings?	
Clarity of minor themes	32	Is there a description of diverse cases or discussion of minor themes?	

Developed from: Tong A, Sainsbury P, Craig J. Consolidated criteria for reporting qualitative research (COREQ): a 32-item checklist for interviews and focus groups. *International Journal for Quality in Health Care*. 2007. Volume 19, Number 6: pp. 349 – 357

Once you have completed this checklist, please save a copy and upload it as part of your submission. DO NOT include this checklist as part of the main manuscript document. It must be uploaded as a separate file.

For peer review only

Supplementary file 2 – Description of CER studies

CER studies are conducted to compare the effectiveness or cost-effectiveness of two or more interventions (treatments or diagnostics) for a specific patient population, to identify which interventions work best for which patient. In a previous study (de Weerdt et al., 2023 currently under submission), we developed claims-based A&F for six CER studies conducted in the Netherlands. These CER studies are described below.

Table. CER studies for which examples of claims-based A&F were developed

CER trial	Trial registration number*	Research question	Patient population	Intervention
1) CAPP	NL9371	What is the most effective treatment for children with complex appendicitis	Children of 0-18yrs with complex appendicitis with/ without infiltrate/abscess	• Operative:<br>○ Laparoscopic<br>○ Open procedure<br>• Conservative
2) Proclion	NA**	What is the most effective treatment for patients with critical ischemia of the legs	Patients of ≥18yrs with critical ischemia of the legs	• Operative<br>○ Laparoscopic<br>○ Open procedure<br>• Conservative
3) DART	NL6201	What is the most effective treatment for elderly with an intra-articular distal radius fracture type C	Patients of ≥65yrs with intra-articular distal radial fracture type C?	• Operative<br>• Conservative
4) MIRA2	NL7817	What is the most effective treatment for woman with heavy menstrual bleeding	Patients of 25-60yrs with heavy menstrual bleeding	• Endometrial ablation<br>• Endometrial ablation combined with hormonal IUD
5) STONE	NL8128	What is the most effective treatment for patients with obstructive kidney stones	Patients of ≥18yrs with obstructive kidney stones	• Double J catheter<br>• Nephrostomy catheter
6) Growth hormone	NL6440	What is effect of discontinuation of growth hormone treatment for children with idiopathic growth hormone deficiency that have normal growth hormone levels mid-puberty	Patients of 10-18yrs with idiopathic growth hormone deficiency	• Continued growth hormone treatment<br>• Discontinued growth hormone treatment

For peer review only - http://bmjopen.bmj.com/site/about/guidelines.xhtml

Supplementary File 3 - Focus group protocol

At the beginning of each focus group rules of conduct were set, the aim of the focus group was explained, and informed consent was repeated. Prior to the start of the actual content of the focus group all researchers and participants were asked to introduce themselves and describe their current employment.

The main research questions was:
What are the perceptions of medical specialists on claims-based Audit & Feedback for QI?

The following sub-questions were discussed during the focus groups:

(1) Are you familiar with A&F and/or with claims-based A&F? What are your perceptions on A&F and/or claims-based A&F?

Participants were presented with visual examples of claims-based A&F for Comparative Effectiveness Research (CER) study:

(2) What are your perceptions on this example of claims-based A&F?
(3) Would you accept this claims-based A&F on CER implementation status?

For peer review only

BMJ Open

Do medical specialists accept claims-based Audit & Feedback for quality improvement? a focus group study

Journal:	*BMJ Open*
Manuscript ID	bmjopen-2023-081063.R1
Article Type:	Original research
Date Submitted by the Author:	10-Jan-2024
Complete List of Authors:	de Weerdt, Vera; Vrije Universiteit Amsterdam, Department of Health Economics; Amsterdam University Medical Centres<br>Ybema, Sierk; Vrije Universiteit Amsterdam, Department of Organization Sciences; Anglia Ruskin University, Department of Organization Sciences<br>Repping, S.; Amsterdam University Medical Centres<br>van der Hijden, Eric; Vrije Universiteit Amsterdam, Department of Health Economics; Zilveren Kruis Health Insurance<br>Willems, Hanna; Amsterdam University Medical Centres, Department of Geriatrics
**Primary Subject Heading**:	Health services research
Secondary Subject Heading:	Health policy, Health informatics, Evidence based practice, Qualitative research
Keywords:	Clinical audit < HEALTH SERVICES ADMINISTRATION & MANAGEMENT, Quality in health care < HEALTH SERVICES ADMINISTRATION & MANAGEMENT, MEDICAL EDUCATION & TRAINING, HEALTH SERVICES ADMINISTRATION & MANAGEMENT, Hospitals

SCHOLARONE™
Manuscripts

I, the Submitting Author has the right to grant and does grant on behalf of all authors of the Work (as defined in the below author licence), an exclusive licence and/or a non-exclusive licence for contributions from authors who are: i) UK Crown employees; ii) where BMJ has agreed a CC-BY licence shall apply, and/or iii) in accordance with the terms applicable for US Federal Government officers or employees acting as part of their official duties; on a worldwide, perpetual, irrevocable, royalty-free basis to BMJ Publishing Group Ltd ("BMJ") its licensees and where the relevant Journal is co-owned by BMJ to the co-owners of the Journal, to publish the Work in this journal and any other BMJ products and to exploit all rights, as set out in our licence.

The Submitting Author accepts and understands that any supply made under these terms is made by BMJ to the Submitting Author unless you are acting as an employee on behalf of your employer or a postgraduate student of an affiliated institution which is paying any applicable article publishing charge ("APC") for Open Access articles. Where the Submitting Author wishes to make the Work available on an Open Access basis (and intends to pay the relevant APC), the terms of reuse of such Open Access shall be governed by a Creative Commons licence – details of these licences and which Creative Commons licence will apply to this Work are set out in our licence referred to above.

Other than as permitted in any relevant BMJ Author's Self Archiving Policies, I confirm this Work has not been accepted for publication elsewhere, is not being considered for publication elsewhere and does not duplicate material already published. I confirm all authors consent to publication of this Work and authorise the granting of this licence.

For peer review only

Do medical specialists accept claims-based Audit & Feedback for quality improvement? a focus group study

V. de Weerdt[1,2], S. Ybema[3] , S. Repping[2,4], E.J.E. van der Hijden[1,5], H.C. Willems[6,7]

[1] Department of Health Economics, Faculty of Business and Economics & Talma Institute, Vrije Universiteit, Amsterdam, the Netherlands

[2] Amsterdam University Medical Centers, University of Amsterdam, Amsterdam, the Netherlands

[3] Department of Organization Sciences, Faculty of Social Sciences & Talma Institute, Vrije Universiteit, Amsterdam, the Netherlands & Anglia Ruskin University, Cambridge UK.

[4] National Healthcare Institute, Diemen, the Netherlands

[5] Zilveren Kruis Health Insurance, Zeist, the Netherlands

[6] Department of Geriatrics, Amsterdam University Medical Centers, University of Amsterdam, Amsterdam, the Netherlands

[7] Leading the Change, Zorgevaluatie Nederland, the Netherlands

Correspondence to:

V. de Weerdt

Department of Health Economics, Faculty of Business and Economics & Talma Institute, Vrije Universiteit, Amsterdam, the Netherlands, de Boelelaan 1105, 1081 HV Amsterdam, the Netherlands

(+31-6-42561363 / v.de.weerdt@vu.nl)

[version 04-01-2024]

For peer review only

Abstract

Objectives Audit & Feedback (A&F) is a widely used quality improvement (QI) intervention in healthcare. However, not all feedback is accepted by professionals. While claims-based feedback has been previously used for A&F interventions, its acceptance by medical specialists is largely unknown. This study examined medical specialists' acceptance of claims-based A&F for QI.

Design Qualitative design, with focus group discussions. Transcripts were analysed using discourse analysis.

Setting and participants A total of five online focus group discussions were conducted between April 2022 and September 2023 with 21 medical specialists from varying specialties (urology; paediatric surgery; gynaecology; vascular surgery; orthopaedics and trauma surgery) working in academic and/or regional hospitals in the Netherlands.

Results Participants described mixed views on using claims-based A&F for QI. Arguments mentioned in favour were (1) A&F stimulates reflective learning and improvement and (2) claims-based A&F is more reliable than other A&F. Arguments in opposition were that (1) A&F is insufficient to create behavioural change (2) A&F lacks clinically meaningful interpretation, (3) claims data is invalid for feedback on QI, (4) claims-based A&F is unreliable, and (5) A&F may be misused by health insurers. Furthermore, participants described several conditions for implementation of A&F which shape their acceptance.

Conclusions Using claims-based A&F for QI is, for some clinical topics and under certain conditions, accepted by medical specialists. Acceptance of claims-based A&F can be shaped by how A&F is implemented into clinical practice. When designing A&F for QI it should be considered whether claims data, as the most resource efficient data source, can be used or whether it is necessary to collect more specific data.

Keywords: Audit & Feedback; Clinical performance feedback; Claims data; Quality Improvement

For peer review only - http://bmjopen.bmj.com/site/about/guidelines.xhtml

STRENGTHS AND LIMITATIONS OF THIS STUDY

- Focus on perspective of medical specialists on claims-based A&F
- Comprehensive findings due to inclusion of medical specialists from varying specialties
- Discourse analysis to create in-depth understanding of participants' views on claims-based A&F
- Reflection on examples of Dutch claims-based A&F and Dutch claims data
- No inclusion of non-surgical specialties

For peer review only

Introduction

Audit and Feedback (A&F) interventions are widely used as a quality improvement (QI) strategy in healthcare.(1) Through A&F, medical professionals receive feedback on their clinical performance, which intends to stimulate them to improve their quality of care.(2–4) Using readily available claims data for A&F aiming to improve quality has large potential benefits, since it can prevent administrative burdens on professionals and limit costs associated with data collection for A&F.(5,6)

However, a review on A&F from registries suggests that claims-based A&F may not be suitable for QI interventions, as use of claims-based A&F is associated with a lower effect on QI as opposed to A&F from quality registries.(7) Healthcare claims data is collected for the purpose of reimbursement of healthcare services. This data contains patients' personal information, a diagnosis code, and billing codes for provided services and medications.(8) As claims data is not collected for clinical decision making or QI, it often lacks clinical detail, information on disease severity, and relevant patient characteristics such as smoking status(5,9,10) Therefore, clinicians may not accept claims-based A&F as "fit-for-purpose" in the context of QI interventions.(11,12)

Research on whether medical specialists accept the use of claims-based A&F in the context of quality improvement is limited. Aim of this study was to examine whether medical specialists accept claims-based A&F intended for QI purposes.

For peer review only

Method

Study Design

We conducted focus groups with medical specialists from varying specialties, to examine their potential acceptance of claims-based A&F. Focus groups were used to allow for discussions between participants on varying perceptions and arguments, thereby creating richness of results.(13)

This study was designed in accordance with the COnsolidated citeria for REporting Qualitative research (COREQ) (Supplementary File 1).(14) All participants provided written informed consent.

Patient or public involvement

Patients or public were not involved in this study.

Contextual background of this study

In a previous study, claims-based A&F was developed in a co-creation approach with six Comparative Effectiveness Research (CER) teams (Supplementary File 2). These CER studies are conducted to identify which interventions are most effective or most cost-effective and the aim is to subsequently implement the CER results in practice to create QI. A&F in this context portrays the level in which CER results are implemented into clinical practice, which is intended to stimulate professionals to implement CER results where necessary.

For peer review only - http://bmjopen.bmj.com/site/about/guidelines.xhtml

108 In the current study, we used this previously developed claims-based A&F to explore whether independent medical specialists, i.e. who were not involved in the A&F development process, accept claims-based A&F for QI purposes.

111

112 ## Participants

113 For each focus group we invited medical specialists working in the specialty of the respective CER study, namely: urology, paediatric surgery, gynaecology, orthopaedics & trauma surgery, and vascular surgery. Recruitment of medical specialists was done between March 2022-June 2023 in two ways: first, we directly invited medical specialists of respective specialisms via e-mail. E-mail addresses were obtained via the CER study teams or via the professional network of the authors. Second, some of the CER study teams also contacted their professional networks via e-mail and/or newsletters. Reasons for doing the research were explained in the recruitment email and at the start of each focus group.

121 In online focus groups, it is recommended to use a smaller number of participants to ensure active participation, thus we aimed for four – six participants per session(15) We aimed to recruit a diverse group of participants in characteristics like gender, years of work experience, geographical region, and type of hospital. We invited 93 medical specialists directly, of which 29 did not respond to the invitation and 10 refused to participate for one of the following reasons: lack of time (N=3), did not consider themselves an expert on the topic (N=5) or no reason (N=2). Of the 54 medical specialists who were interested in participation, 33 were unable to participate on the scheduled dates of the focus groups.

129 In one of the focus groups (DART) three of the eight participants were previously familiar with each other through professional connections, in the other four focus groups none of the participants had previous relationships.

For peer review only

Data collection

Focus groups were held online via Microsoft Teams between April 2022 – September 2023. Focus groups were held during working hours and lasted 60-90 minutes. Researcher HW (MD-PhD, female) or SY (PhD in organization sciences, male) moderated the sessions. Researcher VW (MD-PhD candidate, female) acted as an observer, presented the examples of claims-based A&F for CER trials and asked clarifying questions when necessary. Both moderators had extensive experience with focus group research and moderation of focus groups. The observer had prior experience with qualitative research, but not with moderation of focus groups. Three participants of one focus group (DART) had a previous professional connection to one of the moderators, who had a limited supporting moderation role in this focus group. None of the other participants had previous connections to the researchers. The moderators of the focus groups were not involved in the co-creation study in which the examples of claims-based A&F for CER studies were developed.

Each focus group followed the same protocol (Supplementary File 3). Focus groups consisted of two parts. First, we asked participants whether they were familiar with A&F for QI and whether they were familiar with claims-based A&F to elicit participants general perceptions on A&F and claims-based A&F. Second, we presented participants with the specific examples of claims-based A&F (Supplementary File 4). These examples were visual graphs of claims-based A&F for one specific CER study, which presented the volume of the intervention studied in the CER study, per hospital in the year 2017 (Figure 1). This enabled us to capture participants' perceptions on realistic examples of claims-based A&F.

For peer review only - http://bmjopen.bmj.com/site/about/guidelines.xhtml

Focus groups were audio- or video-recorded and subsequently transcribed ad verbatim. Field notes were taken by the observer during each focus group. Transcripts were not returned to participants. No repeat focus groups were carried out.

Figure 1: hypothetical example

Data analysis

Transcripts were analysed using discourse analysis.(16) Discourse analysis can be useful for creating an in-depth understanding of how people construct contested issues in different ways.(16,17) Discourse analysis can then reveal rhetorical devices people use to make "what they are saying appear factual".

Authors VW and HW independently coded each transcript after a focus group was held, coding was done in Microsoft Word or MAXQDA. To ensure coding reliability, they discussed coding of each transcript and resolved discrepancies through discussion and refined codes. In the fifth and final focus group no new codes were identified, which provided evidence for data saturation. After coding the final focus group, codes were discussed within the wider research team (VW, HW, and SY). The wider research team decided to group codes into arguments in favour and against claims-based A&F. Then, authors VW and HW re-analysed each transcript and labelled all codes as arguments in favour or against claims-based A&F and identified supporting quotes. Again, authors VW and HW discussed labels of each argument and resolved discrepancies through discussion. Subsequently, author VW grouped arguments and supporting quotes. Last, quotes were analysed using a discourse analytic approach to formulate how participants discursively presented their arguments. This was

For peer review only

done by analysing key quotes and singling out the figures of speech and key words participants used in order to make a case for or against claims-based A&F (e.g. ‘it works, because it presents a mirror’). On the basis of this first-order analysis, the underlying discursive strategies which participants adopted (e.g. presenting claims-based A&F as an effective tool for quality improvement) were analysed. These strategies were discussed within the wider research team (VW, HW, SY) until all authors agreed on the formulation of the arguments.

For peer review only

Results

Participants

In total there were 21 participants from six clinical specialties from 17 hospitals in the Netherlands (Table 1). The number of participants per focus group varied from 2-7. Most participants described familiarity with A&F, with 57% describing regular use, and an additional 24% indicating some level of exposure. Conversely, 19% reported no prior experience with A&F. Specifically focusing on claims-based A&F, 38% of participants described regular use, 14% described some level of exposure and 48% described no prior experience with this type of A&F.

Table 1: Participant characteristics

Focus group	Participant	Function	Type of Hospital*	Gender	Familiar with A&F	Familiar with A&F based on claims data
**DART**	D1	Trauma surgeon	Top-clinical	M	Somewhat	No
	D2	Trauma surgeon	Top-clinical	M	Somewhat	Somewhat
	D3	Orthopaedic surgeon, traumatologist	Top-clinical	M	Somewhat	No
	D4	Trauma surgeon	Top-clinical	M	No	No
	D5	Trauma surgeon	Academic	M	Yes	No

	D6	Orthopaedic trauma surgeon	Top-clinical	M	No	Somewhat
	D7	Orthopaedic trauma surgeon	Academic	M	No	No
**STONE**	S1	Urologist	Top-clinical	F	Yes	Yes
	S2	Urologist	Top-clinical	M	Yes	Yes
	S3	Urologist	Academic	M	Yes	Yes
	S4	Urologist	General	F	No	No
**Proclion**	P1	Vascular surgeon	Top-clinical	M	Somewhat	Somewhat
	P2	Vascular surgeon	Top-clinical	M	Yes	No
**MIRA2**	M1	Gynaecologist	General	F	Yes	Yes
	M2	Gynaecologist	Top-clinical	F	Yes	Yes
	M3	Gynaecologist	Academic	M	Yes	Yes
	M4	Gynaecologist	Academic	F	Yes	Yes
	M5	Gynaecologist	General	F	Yes	Yes
**CAPP**	C1	Paediatric surgeon	Academic	M	Yes	No
	C2	Paediatric surgeon	Top-clinical	F	Yes	No
	C3	Paediatric surgeon	Top-clinical	M	Yes	No

* In the Netherlands, general hospitals are non-teaching hospitals. Top-clinical hospitals distinguish themselves from general hospitals by meeting certain criteria, which include teaching programs, an infrastructure for innovation and scientific research, and excelling in areas of clinical care. Academic hospitals are large teaching and research hospitals, which provide tertiary care.(18)

Acceptance of claims-based Audit & Feedback

Most participants were positive in their views on A&F in general, but participants were mixed in their views on claims-based A&F and the specific examples of claims-based A&F for CER studies. While some participants were unambiguously in favour or against claims-based A&F, most participants posed arguments for both sides (Supplementary File 5 and 6). Participants who were familiar with A&F were more positive towards claims-based A&F, than participants who were unfamiliar with A&F. Furthermore, participants who were in favour of A&F for QI, argued their stance depended on how claims-based A&F was implemented in practice.

For peer review only

1. Arguments in favour of claims-based A&F

1.1. A&F stimulates reflective learning and improvement

First, participants who viewed A&F favourably described it as a tool that creates reflective learning and improvement. They believed A&F could contribute to QI by providing feedback on whether CER results are implemented into clinical practice. Participants described the presented visual graphs of claims-based A&F as a "*visually attractive tool*" to initiate change. They believed feedback could stimulate a conversation on change. To explain their positive assessment, they used metaphors to frame A&F as a tool that activates and accelerates change: "*A&F is setting everything in motion*", and as an instrument that stimulates self-critical reflection: "*Showing people a mirror is why it works*", because, as another participant put it: *"By comparing yourself you gain insights into whether you are doing "the right thing""*. Some participants describe they believe A&F is mostly useful for outliers. The imagery some participants used reflected their positive reception of A&F as a tool for change.

1.2 Claims-based A&F is more reliable than other types of A&F

A second argument in favour of claims-based A&F was that it is more reliable than other types of A&F, as claims-data is perceived as more reliable than self-reported data. Participants describe:

> *"If you can get it out of claims data, then you have objective data on your own process, cause otherwise it is dependent on what the person registers and that can be less reliable"*

"In quality-registry data it depends on the administrator whether A&F is accurate, if you can improve that by claims-based A&F, then you have a better and more accurate registration, which gives better A&F"

Here, participants describe self-reported data negatively by labelling it as potentially "less reliable" or "inaccurate", while they describe claims data as "objective" and "more accurate". These participants pose that "objective" and "more accurate" claims data, leads to "better A&F".

1. Arguments against claims-based A&F

1.1. A&F is insufficient to create change of clinical behaviour

A first counterargument to use A&F was that it is insufficient to create change of clinical behaviour. Some participants question the effectiveness of A&F:

"I wonder then if you would have the same data and would show it to hospitals if that would really lead to change of practice. I don't know yet. Maybe they will still think of explanations for why they deviate from the norm"

Another participant describes the insufficiency of A&F as:

"If you get the A&F, you need to act upon it, but you can't reinforce that, so… there is the dilemma: you can show it, but subsequently you want change, but that change depends on individual hospitals, so…".

Keywords are "dilemma" and "reinforce", the speaker hereby assumes that for behavioural change to happen in professionals this must be enforced, which A&F itself does not do. Another participant described how A&F him does not create the intended QI for him, but it only incentivises him to register claims differently:

For peer review only

"If you have the feeling you are doing evidence-based treatments and that all it takes is to register claims codes differently, then you will change claims codes: we are not more catholic than the pope"

Here, the participant used the metaphor "we are not more catholic than the pope" to describe that medical specialists are not going to strictly abide by the rules to achieve the goal set by A&F, but rather find a loophole to meet the goal without having to change their clinical behaviour.

Thus, participants describe the insufficiency of A&F to create QI, by questioning its effectiveness or by contriving explanations for why A&F will not lead to QI.

1.2. Claims-based A&F lacks clinically meaningful interpretation

A second argument against A&F was that participants felt it was not possible to interpret the visual graphs of claims-based A&F in a clinically meaningful way. Participants questioned the benefits of the data: *"Yes, it is a nice overview. Only, ehm yes, what are the consequences of this figures? I can't .. ehm.. I can't see how to translate this to my practice? [...] How do we or the patient benefit from this exactly?"*. Furthermore, some participants stated more strongly that if they could not interpret the data they would not use it: "*I can't explain these results, thus then I would discard the feedback*".

2.3 Claims data gives an invalid representation of clinical reality

The third counterargument was that claims data gives an invalid representation of clinical reality. When presented with the visual graphs of claims-based A&F on CER, for three out of five cases the participants described the visual graphs negatively: "*It is*

For peer review only

comparing apples and oranges" or *"You will receive critic of it is not specific enough thus I can't do anything with it (…) It is caused by that stupid claims system, it is just not designed properly for this"*. Using the metaphor "comparing apples and oranges" shows the participant believes an unjust comparison is made, in this case the claims-based A&F which is not a valid comparison with the CER population. Furthermore, "*not designed … for this*" also describes that the participant believes claims data is used for a purpose (A&F on CER), for which it is not suitable or valid. Use of the word "stupid" also shows the participants' frustration with the claims system. Thus, for three out of five cases, the participants describe the claims-based A&F as invalid feedback on CER.

1.3. Claims data gives unreliable results on the number of performed interventions

A fourth argument against claims-based A&F was that claims data gives unreliable results on the number of performed interventions, thus claims-based A&F is not reliable either. Participants described personal experiences with registration of claims data and with claims-based A&F to illustrate their point:

> "*Claims diagnoses may be inaccurate as they are registered prior to diagnostic interventions and often not adjusted after diagnostics identify an alternative diagnosis*".

> *"In our own registration we have conducted a procedure a 100 times and claims data only shows 90 procedures, then I think where did the other 10 go?"*

Participants emphasized their mistrust of claims data by stating:

> *"Over the years I have started to greatly mistrust claims data"*

For peer review only - http://bmjopen.bmj.com/site/about/guidelines.xhtml

"If you want to pull data from the claims system, the coding can go wrong and then you get a group out of your search which is not representative of what you actually did (…) you need to be very careful with this: you may think you see everything, but you're only using a flashlight in a dark room."

Here participants use the word "greatly" to emphasize their mistrust in claims data and the metaphor "only using a flashlight in a dark room" to illustrate their perception that claims-based A&F can only show a small part of clinical practice, which may lead to incorrect judgements about clinical performance.

Thus, the participants use anecdotal experiences to convey objectiveness in their arguments against claims-based A&F and figures of speech to emphasize their objections of claims-based A&F.

2.5 Claims-based A&F may be misused by health insurers

The last argument against claims-based A&F was that participants feared the role of the health insurer. Participants described that they feel health insurers may have a conflict of interest, such as financial incentives to decrease volumes of care, instead of QI purposes. "*If that is the health insurer who has all that claims data, then they have an instrument to say, maybe on wrong grounds, you operate too much or you operate too little*" and "*or even worse, that the health insurer will say to me, you can't operate anymore, cause that is too expensive*".

Keywords here are "*wrong grounds*" in which the speaker assumes that health insurers make decisions based on wrong grounds and "*even worse*" to emphasize the severity of their argument.

For peer review only

Furthermore, participants describe that they fear the role of health insurers when using claims-based A&F:

"What I fear…. is that it won't be used for the patient, but that it will be used by health insurers to condemn hospitals and that …it is tricky this"

"Yes, I greatly fear the judgement of the health insurer who is just looking over my shoulder, even though they might be right in the end that we overtreated, but yeah"

"There are always fearful images of if we operate 60% and my neighbour only operates 20% of patients that the health insurer will come to me saying "you operate a little much, that is a little bit expensive, are you performing correctly?" So yeah, claims data is always a bit of a tricky thing (…) I think claims data may be a thing we should avoid"

Here, the participants legitimize their objections to claims-based A&F by posing that the role of health insurers is something that should be "feared".

Furthermore, participants describe that they do not want to justify their behaviour to the health insurer, as this leads to less joy in work:

"And if a health insurer says, (…) you have to do it this way or why did you do that, then I have to justify myself to the health insurer, which will make the work less fun"

Thus, the participants use several types of discourse to describe the role of health insurers in claims-based A&F as negative.

For peer review only

1. Implementation of claims-based A&F in practice

1.1. A&F only suitable for topics in which sufficient clinical evidence is available

Participants argue that A&F is only suitable for topics on which scientific evidence is available for what is considered “best practice”. To emphasize their argument, they describe using A&F without scientific evidence as “dangerous”:

> *“For wrist fractures we have long not figured out what is best for the patient, we do have ideas, but we have long not figured it out, so then the use of A&F could be dangerous”*

Another participant describes:

> *“Well, yeah, you should not give A&F on topics of which the truth is unknown yet, so you should not confuse the opinion of the majority with the truth”*

This participant describes scientific evidence as “*the truth*” and poses that A&F on topics without scientific evidence may cause bias in misconceiving the clinical practice of the majority is “*the best*”.

3.2 Distribution through professional association creates support among medical specialists

Furthermore, participants describe that distribution of A&F done via their professional associations, instead of health insurers, is more likely to be accepted:

> *“I do agree that if A&F comes through our professional association or guideline committee and it is included in our own guideline that there will just be much more support for it”*

For peer review only

A participant emphasizes this point by stating:

"Because yes, that (the clinical guideline) is just our bible"

The participant uses the word "bible" to emphasize the value of and support for clinical guidelines of professional associations.

However, other participants state that who distributes the data is unimportant to them:

"As long as we can assume the A&F is valid, then I don't care if the insurer or the hospital distributes it"

3.3 Function of A&F should not be judgement of performance

Lastly, participants argued claims-based A&F should not be used to judge whether clinical performance is "wrong" or "right":

"but to fully say, here we put the division line, these hospitals are doing right and these are doing wrong based on this type of data, we should stay far away from that, I feel"

"We should use A&F, but we should not "name and shame", that is what I want to stay away from"

Here participants' use of the words "division line" and "name and shame" show their negative perceptions on judgement of performance based on A&F.

Discussion

This study examined medical specialists' acceptance of claims-based A&F for QI through focus group discussions, in which participants were presented with examples of claims-based A&F on CER trials. While participants were mostly positive in their views on A&F in

For peer review only

general, they were mixed in their views on claims-based A&F and on the specific examples of claims-based A&F for CER trials. Most participants put forward arguments both in favour and against claims-based A&F. Arguments in **favour** of claims-based A&F were that (1) A&F stimulates reflective learning and improvement and (2) claims-based A&F is more reliable than other A&F. The **opposition** against A&F as a QI tool was articulated in five ways: participants claimed that (1) A&F is insufficient to create change of clinical behaviour; (2) A&F lacks clinically meaningful interpretation; (3); claims data is perceived as invalid for feedback on QI; (4) claims-based A&F is perceived as unreliable and (5) claims-based A&F may be misused by health insurers. Additionally, participants indicated that their acceptance depended on how claims-based A&F would be implemented: (1) there should be sufficient clinical evidence for the topic of A&F; (2) A&F should be distributed by professional societies; and (3) A&F should not be used to impose judgment on clinical performance.

Participants had varying views on the effectiveness of A&F as a tool to create QI. We observed that participants without prior use of A&F were more sceptical of A&F as a tool than participants who had used A&F prior to the focus group. In line with previous research, this suggests being unknown with A&F, makes it unloved.(19,20) While A&F has been used as QI intervention in healthcare for years, studies consistently report physicians' incapability to interpret population-level feedback.(20–23) This leads to negative perceptions regarding the usefulness of A&F, which decreases acceptance of A&F(20) Thus, our findings seem to corroborate earlier findings that physicians should be trained on how to interpret and use A&F for QI.(20,22) However, our findings also suggest that acceptance of A&F does not depend solely on informing and training physicians.

For peer review only - http://bmjopen.bmj.com/site/about/guidelines.xhtml

Data validity and reliability was mentioned by participants as the most important factor to accept claims-based A&F for QI purposes. Again, the participants gave mixed reactions, some viewed claims data as potentially more reliable than self-reported data, while others questioned the reliability of claims data and the validity of claims-based A&F for QI. Studies examining acceptance of A&F consistently report data quality as the main factor for accepting A&F, irrespective of which data source is used for the feedback.(3,22–26) Other studies on claims-based A&F found physicians had mainly negative perceptions on the quality of claims data(11,22,23,27), while we also find positive views. For claims-based A&F, it is important to note that the validity and reliability are dependent on the clinical topic for which A&F is used. For example, claims data captures whether a patient had a wrist fracture, but lacks detail of the location of the fracture. This detail may not be relevant for reimbursement but can be clinically relevant in the context of QI, thereby decreasing the “fit-for-purpose” of using claims data for A&F for QI. The validity or “fit-for-purpose” for A&F on QI is determined by how specific claims data distinguishes clinically relevant patient groups. For some healthcare fields, claims data is more detailed than others, increasing the “fit-for-purpose” of claims-based A&F for QI within these specialties(28). The reliability of claims data may also vary for clinical topics: participants in our study noted that claims data is registered more reliable for larger procedures (e.g., surgeries) as compared to smaller interventions and diagnoses for acute, conservatively treated disorders. Thus, the applicability of claims data for A&F in QI is contingent on the clinical topic.

While lack of data quality is consistently mentioned as a barrier for using A&F, it is important to note that these objections are not only about the data itself, but professionals’ perceptions play a big role in accepting the data as accurate or not. For example, data accuracy is questioned more strongly when A&F shows performance is low or when

For peer review only

performance is judged(3,25) Data accuracy may also be less questioned in A&F interventions that are voluntary and/or physician-led.(20,29)

Participants in our study also suggested to distribute claims-based A&F via professional associations, instead of via health insurers. This is in line with other research, which finds A&F is more accepted and effective if it comes from a colleague instead of an external.(1,3) While endorsement of A&F by professional associations could be beneficial in its acceptance by physicians, studies suggest that A&F is most effective if it is given by a direct colleague.(1,30) Thus, it would be interesting to examine whether endorsement of A&F by professional associations is sufficient to create QI or whether local champions of A&F are necessary to create change.

In our study we only examined medical specialists' acceptance of the data source used for A&F: claims data. However, acceptance of claims data as the source for A&F does not guarantee medical specialists' acceptance of an A&F intervention. This is also dependent on the content of the feedback (e.g. is it timely, personalized); recipient characteristics (e.g. do they have an internal/external locus of control) and the audit method (e.g. was it transparent).(3,22,24,31) One study posed acceptance of A&F is unnecessary and that the goal of changing clinical behaviour can also be achieved by combining A&F with for example financial incentives.(32) However, lack of physicians' acceptance of A&F will likely not create a sustainable QI culture among physicians.

Our study suggests that, under certain conditions, medical specialists can accept claims-based A&F for QI purposes. In the review of van de Veer et al, it is uncertain whether the source of A&F being claims data is the explaining factor for lower effectiveness of the interventions.(7) Even if claims data is not the most effective data source, it is currently the most resource efficient data source for A&F interventions, especially for large-scale

For peer review only

interventions aiming to provide A&F to multiple providers. Thus, policymakers, managers and professionals should weigh whether the clinical topic can be portrayed by claims-based A&F or whether it is necessary to collect more detailed data for A&F aiming to improve quality.

Strengths & weaknesses

This study examined medical specialists' acceptance of claims-based A&F in the context of QI. The first strength of this study is the discourse analysis through which we were able to elicit medical specialists' mixed views on claims-based A&F, which creates a better understanding of medical specialists' acceptance of claims-based A&F and shows that it is not simply the reliability of the data that determines the acceptability of claims-based A&F, but rather physicians' perceptions of its reliability. Second, to the best of our knowledge, this is the first study on claims-based A&F focussing on the perceptions of medical specialists. Another strength of this study is that we used realistic examples of claims-based A&F on varying CER studies and included medical specialists from various clinical specialities. Furthermore, we included both participants who were and were not familiar with A&F and claims-based A&F, which increases the generalizability of our results.

A limitation of this study is that participants in our study reflected on Dutch claims data. As the content of claims data and the accuracy of registration can vary by country, medical specialists in other countries may view claims-based A&F differently. However, our results describe themes which are common across healthcare systems and our results corroborate and detail previous findings described in A&F literature. Secondly, the participants in our focus groups were mainly surgeons, thus it is unclear whether other healthcare professionals put forward additional perspectives on claims-based A&F. Lastly, in some focus groups we

For peer review only

had a limited number of participants which may have decreased the level of discussion between participants. However, we did reach data saturation over all focus groups, thus we do not believe that additional arguments would have been identified with more participants per focus group.

Conclusion

Using claims-based A&F for quality improvement is, for some clinical topics and under certain conditions, accepted by medical specialists. Our study showed that perceptions of medical specialists on A&F in general and claims-based A&F for QI varied, and acceptance of claims-based A&F can be shaped by how A&F is implemented into clinical practice. Currently, claims data is the most resource efficient data source for A&F interventions. Thus, policymakers, managers and professionals should consider whether the clinical topic can be represented by claims-based A&F or whether it is necessary to collect other data for A&F with the aim of improving quality.

For peer review only

References

1. Ivers N, Jamtvedt G, Flottorp S, Young JM, Odgaard-Jensen J, French SD, et al. Audit and feedback: Effects on professional practice and healthcare outcomes. Cochrane Database of Systematic Reviews. 2012;2012(6).
2. Gude WT, Van Engen-Verheul MM, Van Der Veer SN, De Keizer NF, Peek N. How does audit and feedback influence intentions of health professionals to improve practice? A laboratory experiment and field study in cardiac rehabilitation. BMJ Qual Saf. 2017;26(4):279–87.
3. Brown B, Gude WT, Blakeman T, Van Der Veer SN, Ivers N, Francis JJ, et al. Clinical Performance Feedback Intervention Theory (CP-FIT): A new theory for designing, implementing, and evaluating feedback in health care based on a systematic review and meta-synthesis of qualitative research. Implementation Science. 2019;14(1):1–25.
4. Gude WT, Brown B, Van Der Veer SN, Colquhoun HL, Ivers NM, Brehaut JC, et al. Clinical performance comparators in audit and feedback: A review of theory and evidence. Implementation Science. 2019;14(1):1–14.
5. Mckee M. Routine data: A resource for clinical audit ? Quality in Health Care. 1994;2(2):104–11.
6. Solberg LI, Engebretson KI, Sperl-Hillen JM, Hroscikoski MC, O'Connor PJ. Are claims data accurate enough to identify patients for performance measures or quality improvement? The case of diabetes, heart disease, and depression. American Journal of Medical Quality. 2006;21(4):238–45.
7. van der Veer SN, de Keizer NF, Ravelli ACJ, Tenkink S, Jager KJ. Improving quality of care. A systematic review on how medical registries provide information feedback to health care providers. Int J Med Inform [Internet]. 2010;79(5):305–23. Available from: http://dx.doi.org/10.1016/j.ijmedinf.2010.01.011
8. Peek N, Holmes JH, Sun J. Technical challenges for big data in biomedicine and health: data sources, infrastructure, and analytics. Yearb Med Inform. 2014;9:42–7.
9. Powell AE, Davies HTO, Thomson RG. Using routine comparative data to assess the quality of health care: Understanding and avoiding common pitfalls. Qual Saf Health Care. 2003;12(2):122–8.
10. Iezzoni LI. Annals of Internal Medicine Assessing Quality Using Administrative Data Content of Administrative Databases. Ann Intern Med. 1997;127(2):666–74.
11. van Hoof Thomas; Pearson, David; Giannotti, Tierney; Tate, Janet; Elwell, Anne; Barr JM. Lessons Learned from Performance Feedback. Journal for Healthcare Quality. 2001;28(3):20–31.
12. Bonis PAL. Quality incentive payment systems: Promise and problems. J Clin Gastroenterol. 2005;39(4 SUPPL.):176–82.
13. Freitas H, Oliveira M, Jenkins M, Popjoy O. The Focus Group, A Qualitative Research Method. Isrc. 1998;(010298):1–22.
14. Tong A, Sainsbury P, Craig J. Consolidated criteria for reporting qualitative research (COREQ): A 32-item checklist for interviews and focus groups. International Journal for Quality in Health Care. 2007 Dec;19(6):349–57.
15. Lobe B. A new era in focus group research: challenges innovation and practice. Barbour R, Morgan D, editors. London: Palgrave Macmillan; 2017. 1–14 p.
16. Cheek J. At the margins? Discourse analysis and qualitative research. Qual Health Res. 2004;14(8):1140–50.

For peer review only

17. Stevens P, Harper DJ. Professional accounts of electroconvulsive therapy: A discourse analysis. Soc Sci Med. 2007;64(7):1475–86.
18. Samenwerkende Topklinische Ziekenhuizen. Toelating tot de STZ [Internet]. [cited 2024 Jan 2]. Available from: https://www.stz.nl/over-ons/toelating-tot-de-stz/
19. Rouleau G, Reis C, Ivers NM, Desveaux L. Lipstick on a pig : Understanding efforts to redesign audit and feedback reports for primary care . 2022;1–16.
20. Desveaux L, Ivers NM, Devotta K, Ramji N, Weyman K, Kiran T. Unpacking the intention to action gap: a qualitative study understanding how physicians engage with audit and feedback. Implementation Science. 2021;16(1):1–9.
21. N., Ivers, J., Barnsley, R., Upshur, K., Tu, B., Shah, J. G. My approach to this job is. One person at a time: Perceived discordance between population-Level quality targets and patient-Centred care. Canadian Family Physician [Internet]. 2014;60(3):258–66. Available from: http://www.cfp.ca/content/60/3/258.full.pdf+html%5Cnhttp://ovidsp.ovid.com/ovidweb.cgi?T=JS&PAGE=reference&D=emed12&NEWS=N&AN=2014204124
22. Van Braak M, Visser M, Holtrop M, Statius Muller I, Bont J, Van Dijk N. What motivates general practitioners to change practice behaviour? A qualitative study of audit and feedback group sessions in Dutch general practice. BMJ Open. 2019;9(5):1–8.
23. De Bekker PJGM, De Weerdt V, Vink MDH, Van Der Kolk AB, Donker MH, Van Der Hijden EJE. “Give me something meaningful”: GPs perspectives on how to improve an audit and feedback report provided by health insurers - an exploratory qualitative study. BMJ Open Qual. 2022;11(4):1–9.
24. Payne VL, Hysong SJ. Model depicting aspects of audit and feedback that impact physicians’ acceptance of clinical performance feedback. BMC Health Serv Res [Internet]. 2016;16(1):1–12. Available from: http://dx.doi.org/10.1186/s12913-016-1486-3
25. Hysong SJ, Smitham K, SoRelle R, Amspoker A, Hughes AM, Haidet P. Mental models of audit and feedback in primary care settings. Implementation Science. 2018;13(1):1–11.
26. Rouleau G, Reis C, Ivers N, Desveaux L. Characterizing the Gaps Between Best-Practice Implementation Strategies and Real-world Implementation: Qualitative Study Among Family Physicians Who Engaged With Audit and Feedback Reports. JMIR Hum Factors. 2023;10:1–12.
27. Gerteis M, Peikes D, Ghosh A, Timmins L, O’Malley AS, Barna M, et al. Uses and Limitations of Claims-based Performance Feedback Reports: Lessons From the Comprehensive Primary Care Initiative. Journal for Healthcare Quality. 2018;40(4):187–93.
28. Ulyte A, Bähler C, Schwenkglenks M, Von Wyl V, Gruebner O, Wei W, et al. Measuring diabetes guideline adherence with claims data: Systematic construction of indicators and related challenges. BMJ Open. 2019;9(4):1–8.
29. Hysong SJ, Best RG, Pugh JA. Audit and feedback and clinical practice guideline adherence: Making feedback actionable. Implementation Science. 2006;1(1):1–10.
30. Hysong SJ. Meta-Analysis: Audit and feedback features impact effectiveness on care quality. Med Care. 2009;47(3):356–63.
31. Yi SG, Wray NP, Jones SL, Bass BL, Nishioka J, Brann S, et al. Surgeon-specific performance reports in general surgery: An observational study of initial

For peer review only - http://bmjopen.bmj.com/site/about/guidelines.xhtml

implementation and adoption. J Am Coll Surg [Internet]. 2013;217(4):636-647.e1. Available from: http://dx.doi.org/10.1016/j.jamcollsurg.2013.04.040
32. Ilgen DR, Fisher CD, Taylor MS. Consequences of individual feedback on behavior in organizations. Journal of Applied Psychology. 1979;64(4):349–71.

For peer review only

List of abbreviations

- A&F: Audit & Feedback
- QI: Quality Improvement
- CER: Comparative Effectiveness Research

Figure legend

- Figure 1: Hypothetical example of claims-based A&F on CER implementation level per hospital

For peer review only

Declarations

Ethics approval and consent to participate

This research was conducted in accordance with the ethical guidelines of the Research Ethics Review Committee (BETHCIE) of the Faculty of Science, Vrije Universiteit. All participants received an information letter stating the reasons for conducting the research and provided written informed consent for participation in this study and publication of results. Since the research did not fall under the Medical Research Involving Human Subjects Act (WMO), the study protocol was self-checked by the Research Ethics review committee of the Faculty of Science (BETHCIE) from the VU Amsterdam Faculty of Science, stating that the research project did not require further evaluation by the Research Ethics Review Committee of the VU Amsterdam Faculty of Science.

Availability of data and materials

The data generated and analysed during the current study are not publicly available since these include recordings of focus group sessions with individual persons, but transcripts are available from the corresponding author on reasonable request.

Competing interests

The authors declare that they have no competing interests.

Funding

This research was carried out within the Academic Working Place Care Practice and Policy of the Consortium Quality of Care of the Netherlands Federation of University Medical Centers

For peer review only

(NFU), and National Health Care Institute.’ This funder had no role in the design of the study, data collection, data analysis, interpretation of data and writing the manuscript.

Authors' contributions

Researchers HW, VW and EvdH designed the study. Researcher SY was involved in the study for his extensive expertise in qualitative research and discourse analysis. Researcher HW and/or SY moderated the focus groups, researcher VW was present in the focus groups as an observer and presenter. Researcher SR provided overall supervision and was involved in the write-up of this study. All authors were involved in the write-up of this study.

For peer review only

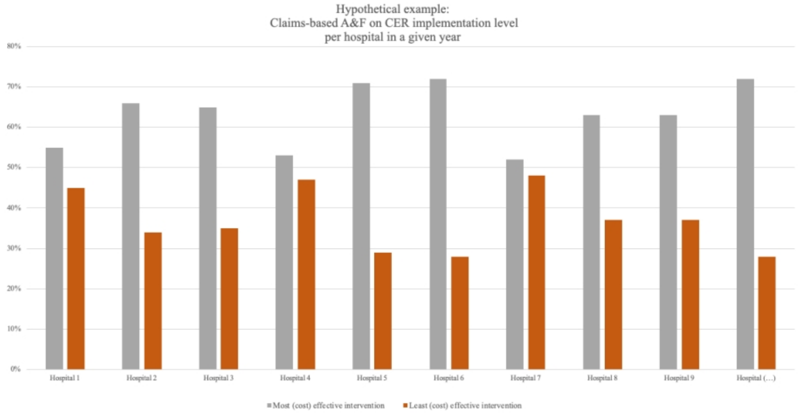

Figure 1 Hypothetical example claims-based A&F on CER implementation level per hospital

338x190mm (108 x 108 DPI)

COREQ (COnsolidated criteria for REporting Qualitative research) Checklist

A checklist of items that should be included in reports of qualitative research. You must report the page number in your manuscript where you consider each of the items listed in this checklist. If you have not included this information, either revise your manuscript accordingly before submitting or note N/A.

Topic	Item No.	Guide Questions/Description	Reported on Page No.
**Domain 1: Research team and reflexivity**			
Personal characteristics			
Interviewer/facilitator	1	Which author/s conducted the interview or focus group?	
Credentials	2	What were the researcher's credentials? E.g. PhD, MD	
Occupation	3	What was their occupation at the time of the study?	
Gender	4	Was the researcher male or female?	
Experience and training	5	What experience or training did the researcher have?	
Relationship with participants			
Relationship established	6	Was a relationship established prior to study commencement?	
Participant knowledge of the interviewer	7	What did the participants know about the researcher? e.g. personal goals, reasons for doing the research	
Interviewer characteristics	8	What characteristics were reported about the inter viewer/facilitator? e.g. Bias, assumptions, reasons and interests in the research topic	
**Domain 2: Study design**			
Theoretical framework			
Methodological orientation and Theory	9	What methodological orientation was stated to underpin the study? e.g. grounded theory, discourse analysis, ethnography, phenomenology, content analysis	
Participant selection			
Sampling	10	How were participants selected? e.g. purposive, convenience, consecutive, snowball	
Method of approach	11	How were participants approached? e.g. face-to-face, telephone, mail, email	
Sample size	12	How many participants were in the study?	
Non-participation	13	How many people refused to participate or dropped out? Reasons?	
Setting			
Setting of data collection	14	Where was the data collected? e.g. home, clinic, workplace	
Presence of non-participants	15	Was anyone else present besides the participants and researchers?	
Description of sample	16	What are the important characteristics of the sample? e.g. demographic data, date	
Data collection			
Interview guide	17	Were questions, prompts, guides provided by the authors? Was it pilot tested?	
Repeat interviews	18	Were repeat inter views carried out? If yes, how many?	
Audio/visual recording	19	Did the research use audio or visual recording to collect the data?	
Field notes	20	Were field notes made during and/or after the inter view or focus group?	
Duration	21	What was the duration of the inter views or focus group?	
Data saturation	22	Was data saturation discussed?	
Transcripts returned	23	Were transcripts returned to participants for comment and/or	

For peer review only

Topic	Item No.	Guide Questions/Description	Reported on Page No.
		correction?	
**Domain 3: analysis and findings**			
Data analysis			
Number of data coders	24	How many data coders coded the data?	
Description of the coding tree	25	Did authors provide a description of the coding tree?	
Derivation of themes	26	Were themes identified in advance or derived from the data?	
Software	27	What software, if applicable, was used to manage the data?	
Participant checking	28	Did participants provide feedback on the findings?	
Reporting			
Quotations presented	29	Were participant quotations presented to illustrate the themes/findings? Was each quotation identified? e.g. participant number	
Data and findings consistent	30	Was there consistency between the data presented and the findings?	
Clarity of major themes	31	Were major themes clearly presented in the findings?	
Clarity of minor themes	32	Is there a description of diverse cases or discussion of minor themes?	

Developed from: Tong A, Sainsbury P, Craig J. Consolidated criteria for reporting qualitative research (COREQ): a 32-item checklist for interviews and focus groups. *International Journal for Quality in Health Care*. 2007. Volume 19, Number 6: pp. 349 – 357

Once you have completed this checklist, please save a copy and upload it as part of your submission. DO NOT include this checklist as part of the main manuscript document. It must be uploaded as a separate file.

For peer review only

Supplementary file 2 – Description of CER studies

CER studies are conducted to compare the effectiveness or cost-effectiveness of two or more interventions (treatments or diagnostics) for a specific patient population, to identify which interventions work best for which patient. In a previous study (de Weerdt et al., 2023 currently under submission), we developed claims-based A&F for six CER studies conducted in the Netherlands. These CER studies are described below.

Table. CER studies for which examples of claims-based A&F were developed

CER trial	Trial registration number*	Research question	Patient population	Intervention
1) CAPP	NL9371	What is the most (cost) effective treatment for children with complex appendicitis: operative or conservative treatment?	Children of 0-18yrs with complex appendicitis with/ without infiltrate/abscess	• Operative:<br>○ Laparoscopic<br>○ Open procedure<br>• Conservative
2) Proclion	NA**	What is the most (cost) effective treatment for patients with critical ischemia of the legs: operative or conservative treatment?	Patients of ≥18yrs with critical ischemia of the legs	• Operative<br>○ Laparoscopic<br>○ Open procedure<br>• Conservative
3) DART	NL6201	What is the most (cost) effective treatment for elderly with an intra-articular distal radius fracture type C: operative or conservative treatment ?	Patients of ≥65yrs with intra-articular distal radial fracture type C?	• Operative<br>• Conservative
4) MIRA2	NL7817	What is the most effective treatment for woman with heavy menstrual bleeding: endometrial ablation or combined treatment of endometrial ablation with hormonal IUD?	Patients of 25-60yrs with heavy menstrual bleeding	• Endometrial ablation<br>• Endometrial ablation combined with hormonal IUD
5) STONE	NL8128	What is the most (cost) effective treatment for patients with obstructive kidney stones: Double J or Nephrostomy catheter?	Patients of ≥18yrs with obstructive kidney stones	• Double J catheter<br>• Nephrostomy catheter
6) Growth hormone	NL6440	What is the most (cost) effective treatment for children with idiopathic growth hormone deficiency that have normal growth hormone levels mid-puberty: continued growth hormone treatment or discontinued growth hormone treatment?	Patients of 10-18yrs with idiopathic growth hormone deficiency	• Continued growth hormone treatment<br>• Discontinued growth hormone treatment

*from the Netherlands Trial Register
** Not registered due to observational study character

For peer review only

Supplementary File 3 - Focus group protocol

At the beginning of each focus group rules of conduct were set, the aim of the focus group was explained, and informed consent was repeated. Prior to the start of the actual content of the focus group all researchers and participants were asked to introduce themselves and describe their current employment.

The main research questions was:
What are the perceptions of medical specialists on claims-based Audit & Feedback for QI?

The following sub-questions were discussed during the focus groups:

(1) Are you familiar with A&F and/or with claims-based A&F? What are your perceptions on A&F and/or claims-based A&F?

Participants were presented with visual examples of claims-based A&F for Comparative Effectiveness Research (CER) study:

(2) What are your perceptions on this example of claims-based A&F?
(3) Would you accept this claims-based A&F on CER implementation status?

For peer review only

Supplementary Files

Claims-based A&F on CER implementation, as shown during focus groups

CER study: DART

CER research question: What is the most (cost)effective treatment for patients ≥65 years old with an intra-articular radial fracture type C: conservative or operative treatment?

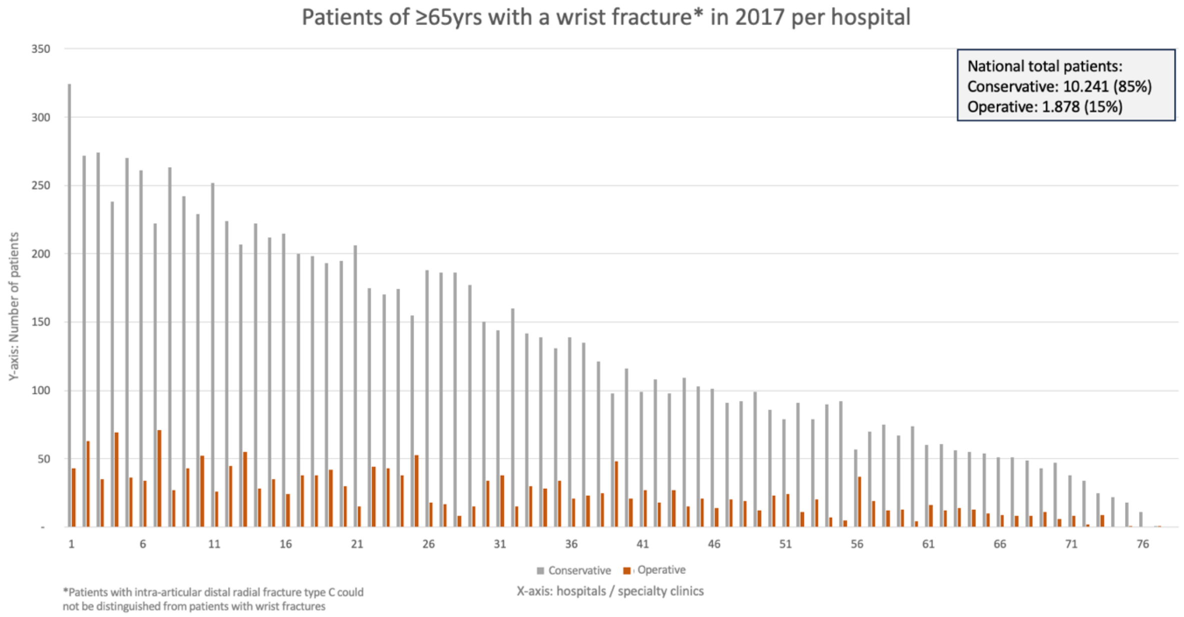

For peer review only - http://bmjopen.bmj.com/site/about/guidelines.xhtml

CER study: STONE

CER research question: What is the most (cost) effective treatment for patients with obstructive kidney stones: Double J or Nephrostomy catheter?

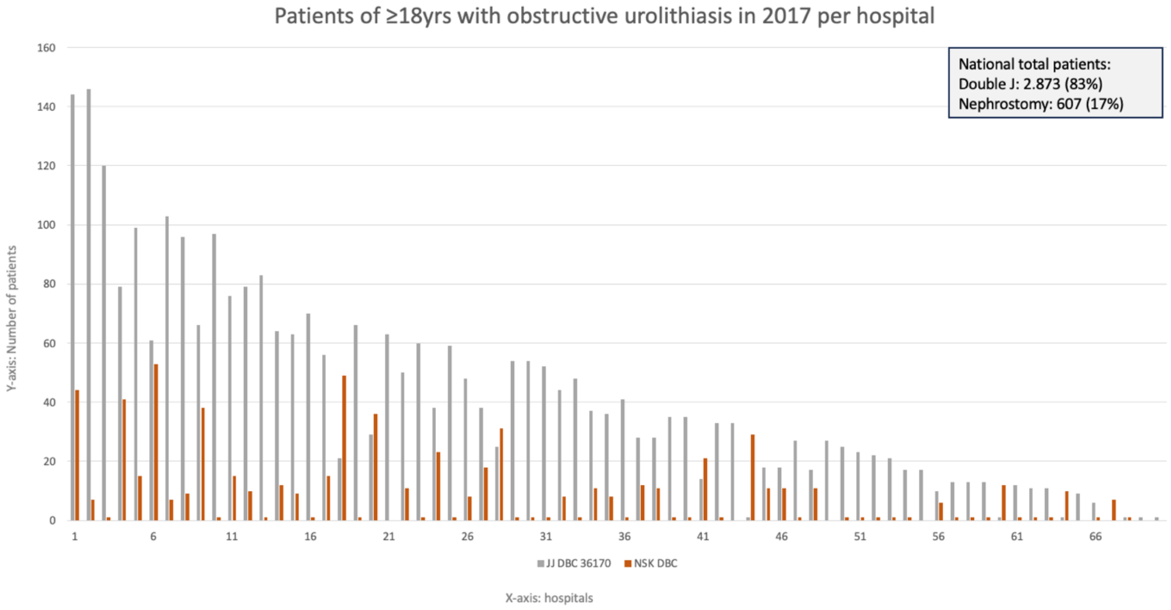

For peer review only

CER study: Proclion

CER research question: What is the most (cost) effective treatment for patients with critical ischemia of the legs: operative or conservative treatment?

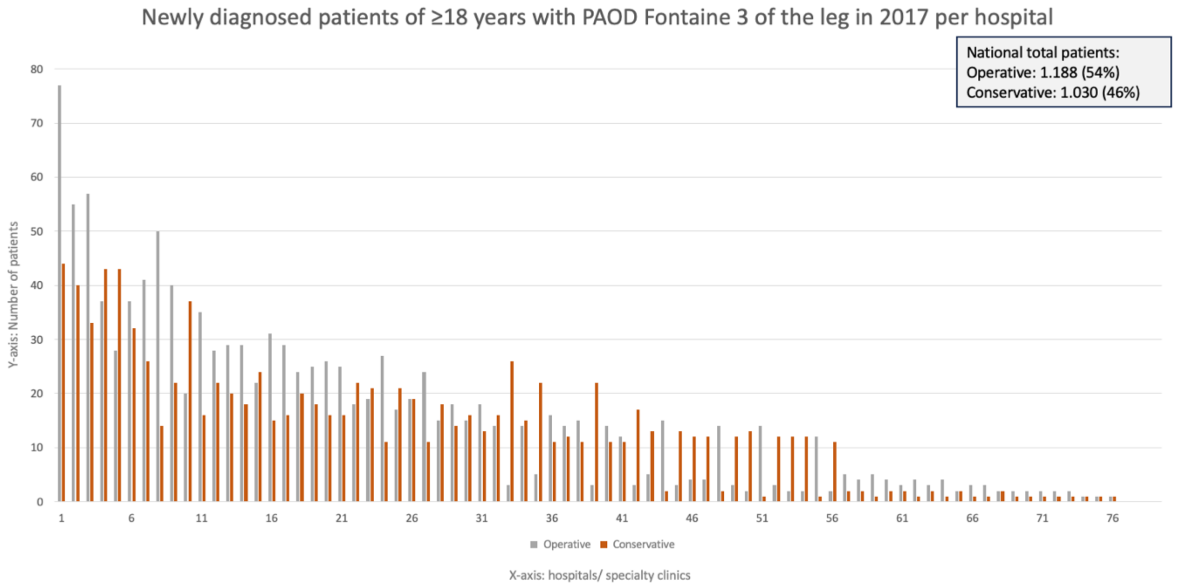

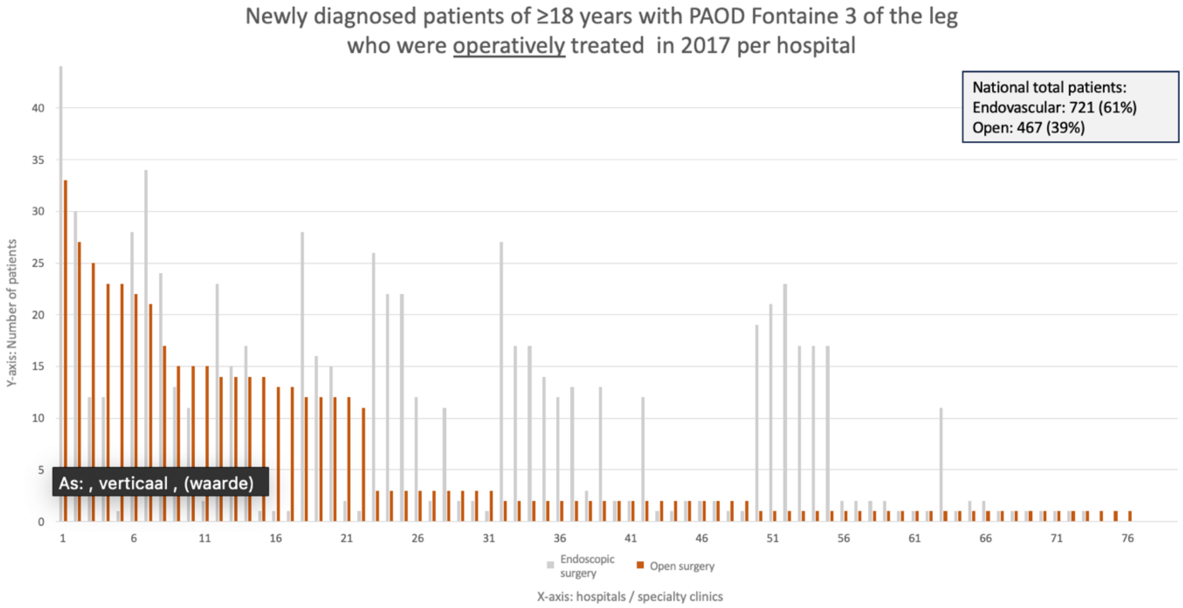

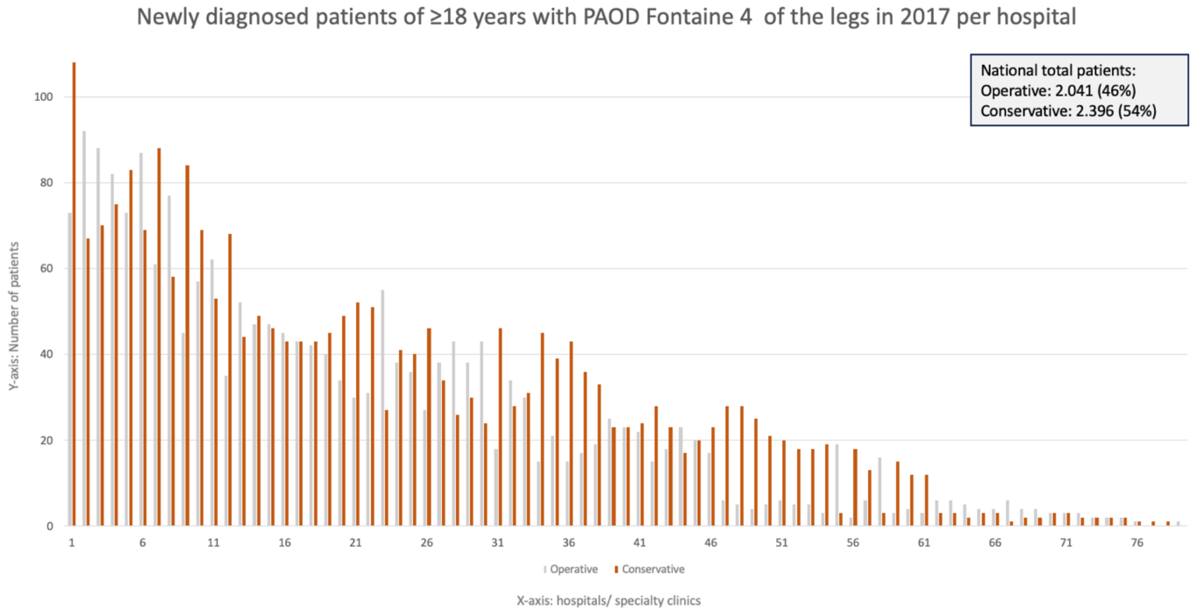
Newly diagnosed patients of ≥18 years with PAOD Fontaine 4 of the legs in 2017 per hospital
National total patients:
Operative: 2.041 (46%)
Conservative: 2.396 (54%)
Y-axis: Number of patients
Operative
Conservative
X-axis: hospitals/ specialty clinics

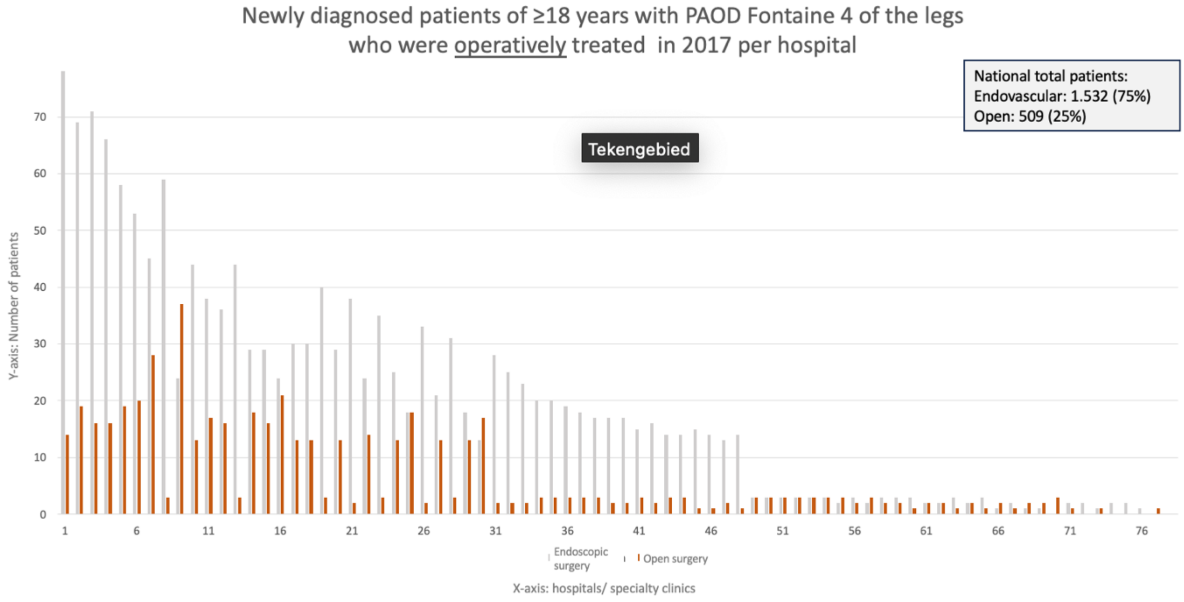
Newly diagnosed patients of ≥18 years with PAOD Fontaine 4 of the legs who were operatively treated in 2017 per hospital
National total patients:
Endovascular: 1.532 (75%)
Open: 509 (25%)
Tekengebied
Y-axis: Number of patients
Endoscopic surgery
Open surgery
X-axis: hospitals/ specialty clinics

4

CER study: MIRA2

CER research question: What is the most (cost) effective treatment for woman with heavy menstrual bleeding: endometrial ablation or combined treatment of endometrial ablation with hormonal IUD?

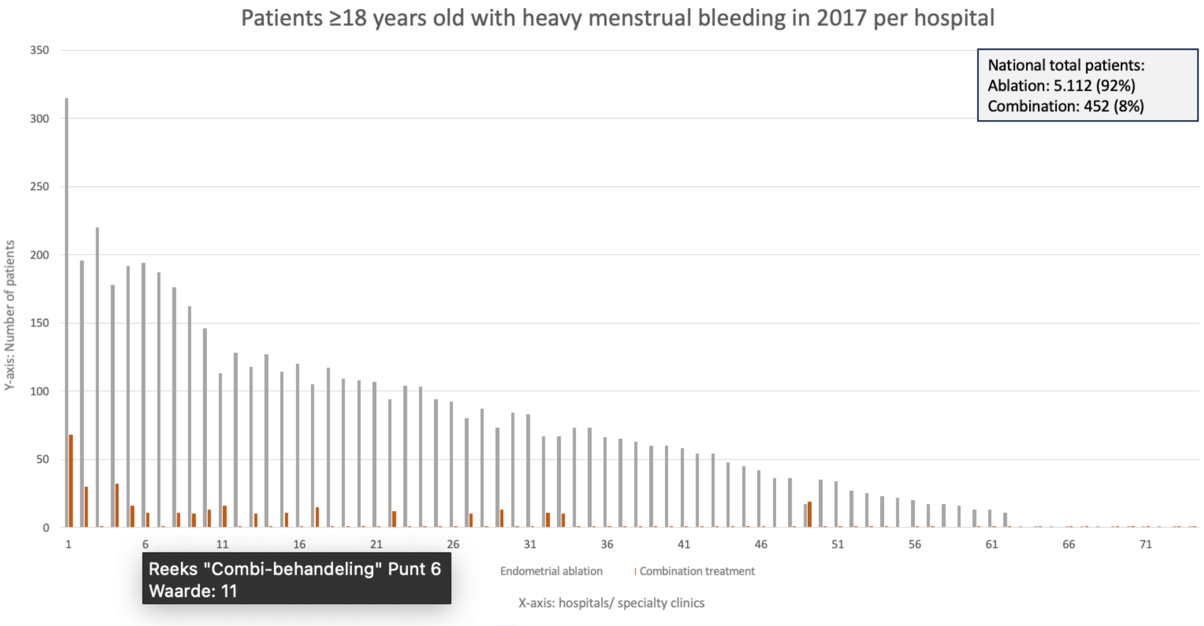

review only

CER study: CAPP

CER Research Question: What is the most (cost) effective treatment for children with complex appendicitis?

- Is laparoscopic or open appendectomy most (cost) effective for children <18 years old with complex appendicitis without abscess/mass formation?
- Is conservative or operative treatment most (cost) effective for children <18 years old with complex appendicitis with abscess/mass formation?

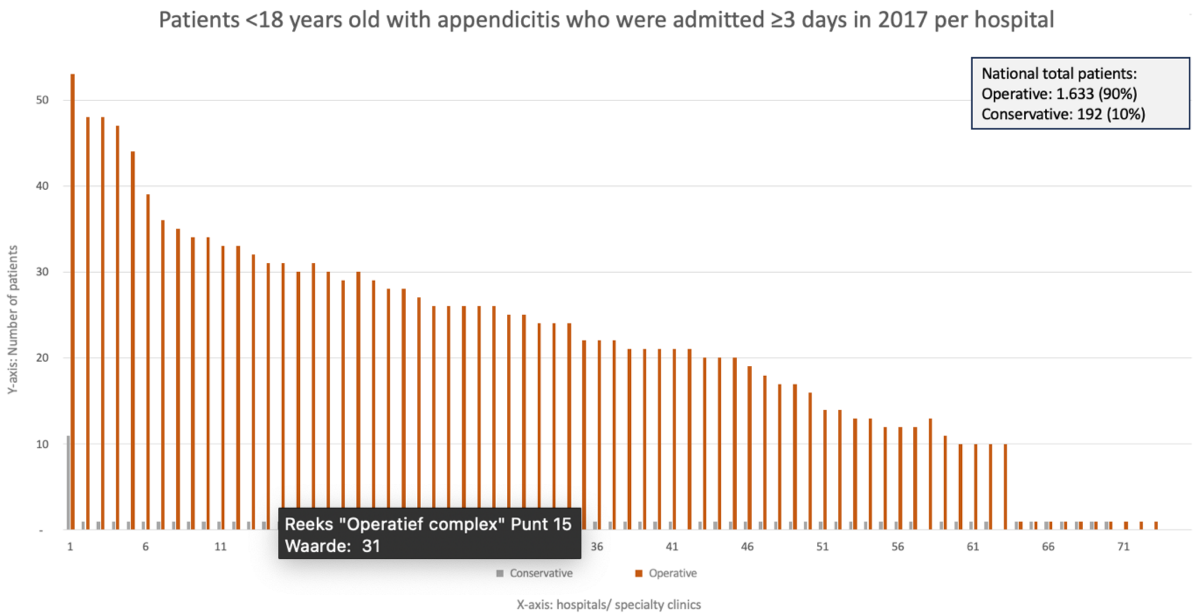

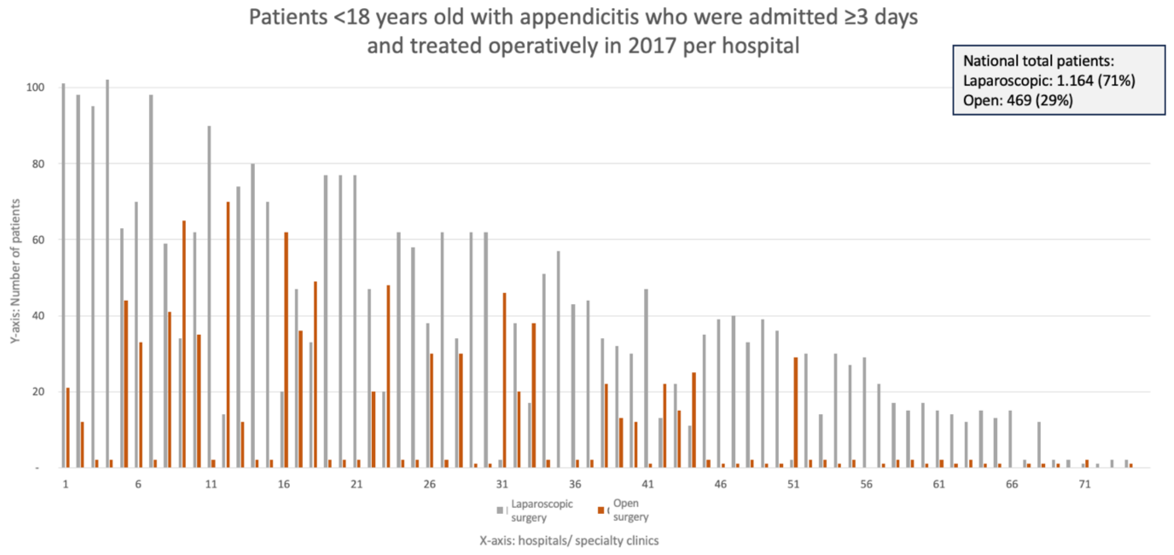

For peer review only - http://bmjopen.bmj.com/site/about/guidelines.xhtml

Table 2: Participants attitudes towards A&F

Focus group	Participant	Attitude towards A&F*	Attitude towards claims-based A&F	Attitude towards A&F developed for this CER study
**DART**	D1	+/-	+/-	-
	D2	+/-	+/-	NS
	D3	+	-	-
	D4	+	+/-	NS
	D5	+	+	NS
	D6	+/-	+/-	+/-
	D7	+/-	+/-	-
**STONE**	S1	+	+	+
	S2	-	-	+/-
	S3	+	+	+
	S4	NS	NS	-
**Proclion**	P1	+/-	-	-
	P2	+/-	-	-
**MIRA2**	M1	+	+/-	-
	M2	+	NS	-
	M3	NS	-	-
	M4	+	NS	-
	M5	+	-	-
**CAPP**	C1	+	+/-	+/-
	C2	+	+/-	+
	C3	+	+	+

* (+)=positive attitude, (+/-)=mixed attitude, (-)=negative attitude, NS=not specified

For peer review only

Table 3. Results discourse analysis

		Quotes	Keywords or Figures of speech	Discourse	Argument
**In favour of claims-based A&F**	1.1	*"I think this (claims-based A&F) is a visual attractive thing to set everything in motion (…) and to stimulate implementation of result results, I do think it helps"*	Sets in motion	A&F gives insight in own performance and thereby creates action	1.1 A&F stimulates reflective learning and improvement
		"Presenting a mirror is why it works"	Comparison of A&F to a mirror		
		"A&F is useful to start a conversation for change, A&F is the first step to initiate change"	Start<br>First step		
	1.2	*"Claims data can give a more reliable portrayal of clinical practice, thus then you get reliable A&F: now it is dependent on the administrator whether A&F is accurate, if you can enhance that through A&F based on data of other sources, then you have a better and more accurate registration which gives better A&F"*	Reliable<br>Accurate	Claims-data is more reliable than self-reported data, thus claims-based A&F is more reliable than other types of A&F	1.2 Claims-based A&F is more reliable than other types of A&F
		"If you can get it out of claims data, then you have objective data on your own process, because otherwise it is dependent on what the person registers and that can be less reliable"	Objective data		
**Opposing claims-based A&F**	2.1	*"I wonder then if you would have the same data and would show it to hospitals if that would really lead to change of practice. I don't know yet. Maybe they will still think of reasons to deviate from the norm"*	Wonder if that would lead to change	Questioning the effect of claims-based A&F	2.1 Claims-based A&F is insufficient to create change of clinical behaviour
		"If you get the A&F, you need to act upon it, but you can't reinforce that, so… there is the dilemma: you can show it, but subsequently you want change, but that change depends on individual hospitals, so…".	Can't reinforce (…) action<br>Dilemma	Questioning the effect of claims-based A&F	
		"if you have the feeling you are doing evidence-based treatments and that all it takes is to register claim codes differently, then you will change claims code, we are not more catholic than the pope"	Change claims code<br>Metaphor: not more catholic than the pope	Posing claims-based A&F leads to different registration of claims-codes, not change of clinical behaviour	
	2.2	[when shown example of claims-based A&F] *"I can't explain it, thus then I would discard the graph"*	Can't explain, thus discard	A&F has no use if results are not explained	2.2 Claims-based A&F lacks clinically

For peer review only

				meaningful interpretation
	[when shown example of claims-based A&F] *“Yes, it is a nice overview. Only, ehm yes, what are the consequences of this figures? I can’t .. ehm.. I can’t see how to translate this to my practice? [...] How do we or the patient benefit from this exactly?”.*	How do we or the patient benefit?	Questioning the relevance of the A&F	
2.3	*“You need to be very careful, that you think you see everything, but you are shining with a flashlight in a dark room”*	Metaphor: “shining with a flashlight in a dark room”	A&F shows only a small piece of the picture	2.3 Claims-data gives an invalid representation of clinical reality
	[when shown example of claims-based A&F] *“This is comparing apples and oranges”*	Metaphor: comparing apples and oranges”	Data does not reflect clinical reality	
2.4	*“In the past we have done a lot with it: is the practice variation the same as registration variation? And we have discovered that it just does not match 1:1 (…) that is the misery, the claims data I have come to greatly mistrust over the years, it is often not about the actual treatment that was performed”*	Practice variation is not the same as registration variation<br>Greatly mistrust claims data	Numbers of intervention performed in claims data do not match numbers of interventions in reality	2.4 Claims-data gives unreliable results on number of interventions performed
	“in our own registration I performed a procedure 100 times, in claims data I only see 90, then I think, where did the other 10 go?”	Anecdotal experience<br>Wrong numbers in claims data		
2.5	*“If that is the health insurer who has all that claims data, then they have an instrument to say, maybe on wrong grounds, you operate too much or you operate too little” (…) “or even worse, that the health insurer will say to me, you can’t operate anymore, cause that is too expensive”.*	If health insurer has all that data<br>Wrong grounds	Health insurers can use claims-based A&F for wrong goals	2.5 Claims-based A&F may be misused by health insurers
	“What I fear…. is that it won’t be used for the patient, but that it will be used by health insurers to condemn hospitals and that … tricky this”	Fear<br>Not used for the patient<br>Used to condemn hospitals<br>Tricky		
	“Yes, I greatly fear the judgement of the health insurer who is just looking over my shoulder, even though they might be right in the end that we overtreated, but yeah”	Fear judgement of health insurer<br>Looking over my shoulder		
	“There are always fearful images of if we operate 60% and my neighbour only operates 20% of patients that the health insurer will come to me saying “you	Fearful images the health insurer will say you operate too much		

For peer review only

		operate a little much, that is is little bit expensive, are you performing correctly?” So yeah claims data is always a bit of a tricky word (…) I think claims data may be a word we should avoid”	Tricky this		
		“Let me put it this way if a big health insurer says you have to do it this way of why did you do that, then I have to justify myself to the health insurer which will make the work less fun”	Health insurers says you have to do it this way<br>Justify myself		
**Conditions for implementation of claims-based A&F**	3.1	*“For wrist fractures we have long not figured out what is best for the patient, we do have ideas, but we have long not figured it out, so then the use of A&F could be dangerous”*	Have not figured out what is best for the patient<br>Dangerous	Scientific evidence on what is best treatment needs to be clear before A&F can be used	3.1 A&F can only be used if scientific evidence exists for best practice
		“Well, yeah, you should not give A&F on topics of which the truth is unknown yet, so you should not confuse the opinion of the majority with the truth”	Should not confuse opinion of the majority with the truth		
	3.2	*“I do agree with respondent (xxx) that if (the A&F) comes from our professional society or via the guideline committee, and the evidence is included in our guideline, that that gives much more support because yes, that (the clinical guideline) is just our bible”*	More support if A&F comes from professional society<br>Clinical guideline is our bible	Distribution through professional society gives more support for A&F	3.2 Distribution through professional society gives more support for A&F
		“Distribution through professional society gives more support for A&F”	Don’t care who distributes		
	3.3	*“But to fully say, here we put the division line, these hospitals are doing right and these are doing wrong based on this type of data, we should stay far away from that, I feel”*	Division line	A&F is not suitable to make judgements on “right or wrong”	3.3 A&F should not be used for judgement
		“We should use A&F, but we should not “name and shame”, that is what I want to stay away from”	Name and shame		

For peer review only
